# Hydrodynamic shear stress promotes epithelial-mesenchymal transition by downregulating ERK and GSK3β activities

**DOI:** 10.1186/s13058-018-1071-2

**Published:** 2019-01-16

**Authors:** Hye Yeon Choi, Gwang-Mo Yang, Ahmed Abdal Dayem, Subbroto Kumar Saha, Kyeongseok Kim, Youngbum Yoo, Kwonho Hong, Jin-Hoi Kim, Cassian Yee, Kyung-Mi Lee, Ssang-Goo Cho

**Affiliations:** 1 Department of Stem Cell & Regenerative Biotechnology and Incurable Disease Animal Model and Stem Cell Institute (IDASI), Konkuk University, 120 Neungdong-ro, Gwangjin-gu, Seoul, 05029 Republic of Korea; 2 Department of Biochemistry and Molecular Biology, Korea University College of Medicine, 26-1 Anam-dong, Sungbuk-gu, Seoul, 02841 Republic of Korea; 3 Department of Surgery, Konkuk University School of Medicine, Seoul, 05030 Republic of Korea; 4 Department of Melanoma Medical Oncology, MD Anderson Cancer Center, Houston, TX 77054 USA

**Keywords:** Tumor-initiating cells, Hydrodynamic shear stress, ROS/NO, EMT/MET, ERK-GSK3β

## Abstract

**Background:**

Epithelial-mesenchymal transition (EMT) occurs in the tumor microenvironment and presents an important mechanism of tumor cell intravasation, stemness acquisition, and metastasis. During metastasis, tumor cells enter the circulation to gain access to distant tissues, but how this fluid microenvironment influences cancer cell biology is poorly understood.

**Methods and results:**

Here, we present both in vivo and in vitro evidence that EMT-like transition also occurs in circulating tumor cells (CTCs) as a result of hydrodynamic shear stress (+SS), which promotes conversion of CD24^middle^/CD44^high^/CD133^middle^/CXCR4^low^/ALDH1^low^ primary patient epithelial tumor cells into specific high sphere-forming CD24^low^/CD44^low^/CD133^high^/CXCR4^high^/ALDH1^high^ cancer stem-like cells (CSLCs) or tumor-initiating cells (TICs) with elevated tumor progression and metastasis capacity in vitro and in vivo. We demonstrate that conversion of CSLCs/TICs from epithelial tumor cells via +SS is dependent on reactive oxygen species (ROS)/nitric oxide (NO) generation, and suppression of extracellular signal-related kinase (ERK)/glycogen synthase kinase (GSK)3β, a mechanism similar to that operating in embryonic stem cells to prevent their differentiation while promoting self-renewal.

**Conclusion:**

Fluid shear stress experienced during systemic circulation of human breast tumor cells can lead to specific acquisition of mesenchymal stem cell (MSC)-like potential that promotes EMT, mesenchymal-epithelial transition, and metastasis to distant organs. Our data revealed that biomechanical forces appeared to be important microenvironmental factors that not only drive hematopoietic development but also lead to acquisition of CSLCs/TIC potential in cancer metastasis. Our data highlight that +SS is a critical factor that promotes the conversion of CTCs into distinct TICs in blood circulation by endowing plasticity to these cells and by maintaining their self-renewal signaling pathways.

**Electronic supplementary material:**

The online version of this article (10.1186/s13058-018-1071-2) contains supplementary material, which is available to authorized users.

## Background

Cancer metastasis involves a series of sequential steps including epithelial mesenchymal transition (EMT) of primary tumor cells into tumor-initiating cells (TICs) and their intravasation into the bloodstream as circulating-tumor cells (CTCs) [1]. Extravasation of CTCs at distant sites, where mesenchymal epithelial transition (MET) culminates, allows their proliferation as epithelial metastatic deposits [2]. CTCs have been detected in the majority of patients with breast cancer, where they represent cancer stem-like cells (CSLCs) or TICs captured as they transit through the bloodstream [3, 4].

Although the concept “seed and soil” or “TICs and hypoxic stromal niche” has been the prevailing determinant of EMT transition and metastasis of breast cancer [5], proliferating tumor cells near the periphery of solid tumor mass are prone to translocation into nearby blood vessels due to the nature of loose mosaic vessels that are formed within solid tumors, facilitated by chemotherapy-induced destruction of extracellular matrix (ECM) [6–8] and a remodeled tumor vasculature [9, 10]. Once translocated into blood vessels, tumor cells present in blood vessels are continually exposed to severe shear stress (SS) under normoxic conditions, and subjected to cell cycle arrest due to hypoxic-to-normoxic transition and apoptosis from the absence of cell-ECM interaction and tumor growth factors. Therefore, only those CTCs acquiring EMT potential during systemic circulation can survive and metastasize to distant sites.

Typical SS is 1–6 dyne/cm^2^ for venous circulation and 15–20 dyne/cm^2^ for arterial circulation [11, 12]. It is gradually appreciated that the mechanical properties of both the tumor microenvironment and cancer cells themselves play a significant role in tumor progression and metastasis [13, 14]. SS exerts prominent force on normal cells and its effects on blood cells, endothelial cells, smooth muscle cells, and epithelial cells, have been broadly studied [15–17]. However, much less is known about the SS effect on intravascular tumor cells of epithelial origin.

Recent studies have demonstrated that SS exerted on circulating hematopoietic stem cells (HSCs) can trigger the onset of definitive hematopoiesis and embryogenesis [18]. Since cancer metastasis begins by the intravasation of tumor cells into the circulation [19–22], we hypothesized that hydrodynamic shear stress (+SS) given to tumor cells could also trigger stress-dependent transcriptional changes that allow transition of CTCs into CSLCs/TICs. Here, we provide both in vivo and in vitro evidence that the conversion from epithelial tumor cells into CSLCs/TICs can occur within blood vessels, due to +SS experienced during systemic circulation without additional requirement for growth factors or a hypoxic stromal niche.

## Methods

### Cell culture and chemicals

Breast tumor tissue specimens were collected from patients who underwent mammotomy biopsy of breast tumors at the Breast Cancer Center of Konkuk University Hospital, with Institutional Review Board (IRB, KUH 1020003) approval. Breast tumor tissues from patients with stage III breast cancer treated with neoadjuvant chemotherapy (four cycles of preoperative doxorubicin and cyclophosphamide) were washed with PBS (GE Healthcare Hyclone) and incubated with type IV collagenase (Sigma-Aldrich) for 20 min at room temperature. A single-cell suspension was obtained by filtering the supernatant through a 100-μm cell strainer (BD Bioscience) and was cultured in DMEM (GE Healthcare Hyclone) supplemented with 10% fetal bovine serum (FBS; GE Healthcare Hyclone) and 1% P/S (100 U/ml penicillin/streptomycin; GE Healthcare Hyclone) at 37 °C with 5% CO_2_. Unless stated otherwise, cells were cultured on the adherent tissue culture plates (Nunc). The study also included breast tumor tissues obtained from 45 patients for CSLC analysis (Additional file 1: Table S1). MDA-MB231 cells (American Type Culture Collection (ATCC)) were cultured in RPMI-1640 medium (Gibco-BRL), supplemented with 10% FBS and 1% P/S at 37 °C with 5% CO_2_. The breast cancer cells (MCF7) were cultured in DMEM supplemented with 10% FBS and 1% P/S at 37 °C with 5% CO_2_. The hepatic cancer (SNU447 and HepG2), colon cancer (HCT116 and HT29), and pancreatic cancer (Panc2 and Capan1) cell lines were cultured in DMEM supplemented with 10% FBS and 1% P/S at 37 °C with 5% CO_2_. Cell viability was assessed by performing trypan blue exclusion assay and cells were counted with a hemocytometer. Doxorubicin (Sigma-Aldrich) and paclitaxel (Calbiochem) were dissolved in dimethyl sulfoxide (DMSO) (Sigma-Aldrich). BIO (GSK3β inhibitor), PD98059 (mitogen-activated protein kinase (MAPK) kinase (MEK) inhibitor), SB203580 (p38 MAPK inhibitor), and SP600125 (c-Jun N-terminal kinase (JNK) inhibitor) were purchased from EMD Millipore.

### Application of hydrodynamic shear stress in vitro

Hydrodynamic shear stress (+SS), which mimicked SS exerted during blood circulation in vivo, was applied on cell cultures using an orbital shaker (DAIHAN Scientific, Model SHO-1D) [23–27]. Tumor cells in DMEM containing 10% FBS and 1% P/S were placed in non-coated Petri dishes (Nunc) and cultured on top of an orbital shaker at 60 rpm for 10 days. Medium was changed every 2 days. After 3, 5, 7, and 10 days, the suspended and adherent cells were harvested separately. SS applied on cell culture plates was estimated using the formula:

$$ {\tau}_{max}=\mathrm{a}\sqrt{\upeta \uprho \left(2\uppi \mathrm{f}\right)3} $$

where a is the orbital radius of rotation of the shaker, ρ is the density of the culture medium (0.9973 g/ml), η is the viscosity of the medium (0.0101 P measured with a viscometer), and f is the frequency of rotation (rotation/sec) [23]. Alternatively, confluent tumor cells, cultured in DMEM supplemented with 10% FBS and 1% P/S in non-coated Petri-dishes, were exposed to flow in an in vitro fluid SS device, the cone-and-plate viscometer. Unidirectional steady flows at SS of 20 dyne/cm^2^ (270 rpm) were applied to the cells to investigate the effect of laminar shear stress (LSS) [23] and bi-directional disturbed flows at 67 rpm corresponding to SS of 5 dyne/cm^2^ were applied to the cells to examine the effect of oscillatory shear stress (OSS) [23].

### Sphere formation assay

To evaluate the self-renewal and differentiation capacity of tumor cells at the single-cell level in vitro, 10,000 tumor cells were cultured in sphere-forming medium ((SFM): DMEM/F12 (Gibco-BRL) supplemented with B27-supplement (dilution, 1:50; Invitrogen), 20 ng/ml epidermal growth factor (EGF; Sigma-Aldrich), 10 μg/ml insulin (Sigma-Aldrich), and 0.4% BSA (bovine serum albumin; Sigma-Aldrich)] for 3 days in non-coated Petri dishes, as described previously [28]. The number of spheres was assessed by trypan blue exclusion staining and counted with a hemocytometer.

### Tumor implantation and metastasis studies

All animal experiments were performed in accordance with the Guide for the Care and Use of Laboratory Animals under approval by t he Institutional Animal Care and Use Committee (IACUC) of Konkuk University (KU11039 and KU12076).

For preparation of the bio-fluorescent green fluorescent protein (GFP)^+^ MDA-MB231, CT-PC (breast cancer cells derived from chemotherapy-treated patients), or CT + SS (CT-PCs generated upon +SS) cells, HEK293T cells were first transfected with the GFP-expressing lentiviral vector, pCD521A (pCDH-EF1-T2A-copGFP), and viral packaging vectors using the calcium phosphate transfection protocol. After 5 days, the virus was harvested and concentrated using an ultracentrifuge (Optima L-90 K, Beckman Coulter Inc.). Then, MDA-MB231, CT-PC, or CT + SS cells were infected with lentivirus and incubated overnight at 37 °C with 5% CO_2_. To measure virus titer, we used the equation as previously described [29]:

$$ \mathrm{IU}/\mathrm{ml}=\left[\left\{\left(\#\mathrm{Cells}\ \mathrm{at}\ \mathrm{starting}\ \mathrm{time}\right)\times \left(\mathrm{Dilution}\ \mathrm{factor}\right)\times \left(\mathrm{Percentage}\ \mathrm{of}\ \mathrm{in}\mathrm{fection}\right)\right\}/\left(\mathrm{Volume}\ \mathrm{virus}\ \mathrm{solution}\ \mathrm{added}\ \mathrm{expressed}\ \mathrm{in}\ \mathrm{ml}\right)\right] $$

Viral injection efficiency was calculated to be < 95%, and ~ 7.8 × 10^8^ IU/ml viral particles were used to prepare bio-fluorescent GFP^+^ cells. The bio-fluorescent GFP^+^ cells (2 × 10^5^ cells) were injected into the mammary fat pads or left ventricle of female immune-deficient (NOD-SCID) or nude mice. Control group mice were injected with 100 μl PBS only. At 1 or 2 days after injection, GFP+ fluorescence was measured using the Xenogen IVIS SPECTRUM (Perkin Elmer). At 2 and 28 days after injection, whole blood was collected using heparin-coated capillary tubes for further analysis. Lysis of erythrocytes (red blood cells (RBC)) was performed as previously reported [30]. Cells from the tibia were isolated using 8 ml of ice-cold PBS and were RBC-lysed with ammonium-chloride-potassium (ACK) lysing buffer (1.5 M NH_4_Cl (Sigma-Aldrich), 100 mM KHCO_3_ (Sigma-Aldrich), 10 mM EDTA (Gibco-BRL), pH 7.2). After RBC lysis, the GFP^+^ cells in the blood or tibia were sorted for GFP+ populations using FACSVantage™ flow cytometer (Becton Dickinson). Cells from the mammary fat pad were excised into small pieces of mammary gland and digested using 3 ml DMEM/F12 with 0.5% collagenase (Sigma-Aldrich) and 0.5% hyaluronidase (Sigma-Aldrich). After 3 h, the tissues were washed three times in PBS containing 1% FBS. Cells were collected by centrifugation and plated on 0.03% collagen-coated cell culture plates in DMEM supplemented with 10% FBS for further RNA and protein analysis. Tumor volumes were calculated as:

$$ \mathrm{Volume}\ \left({\mathrm{mm}}^3\right)=\mathrm{lLength}\times {\mathrm{width}}^2\times 0.5. $$

For serial tumor re-transplantation assay, 100–10,000 (10^2^–10^4^) tumor cells were implanted subcutaneously onto NOD/SCID mice. After tumors were formed, tumors from the first xenografts were excised from the primary recipients, dissociated into a single-cell suspension, grown in adherent culture conditions on gelatin-coated tissue culture plates for approximately 1 week, and then re-injected into secondary mouse recipients.

For orthotopic transplantation analysis of CT-PC or CT + SS, cells (each 1 × 10^4^ cells) were grafted into the mammary fat pad of female NOD/SCID mice. At 4 or 8 weeks after injection, GFP^+^ fluorescent images were obtained using Xenogen (Caliper life science) IVIS SPECTRUM and the GFP^+^ cells in the blood were sorted and counted for GFP^+^ populations using the FACSVantage™ flow cytometer.

To determine the serum ionized calcium levels as a measure of metastasis, ionized calcium was measured using an ABL800 analyzer (Radiometer). Serum albumin was detected using an Olympus AU4500 (Olympus) automatic chemical analyzer. Serum calcium was calculated using the Payne formula:

$$ \mathrm{Corrected}\kern0.17em \mathrm{calcium}\;\left(\mathrm{mg}/\mathrm{dl}\right)=\mathrm{Total}\kern0.17em \mathrm{calcium}\;\left(\mathrm{mg}/\mathrm{dl}\right)+0.8\times \left[4-\mathrm{Albumin}\;\left(\mathrm{g}/\mathrm{dl}\right)\right]. $$

### Immuno-histochemical analysis

For examination, slides were stained with hematoxylin and eosin (H&E; Santa Cruz Biotechnology) according to standard protocols. For immunostaining, sections were incubated with anti-N-CADHERIN (1:500; Santa Cruz Biotechnology), anti-E-CADHERIN (1:500; Santa Cruz Biotechnology), or anti-TWIST (1:500; Santa Cruz Biotechnology) antibody in 0.5% blocking buffer (normal goat serum; Vector Laboratory). The sections were washed and incubated with anti-mouse or anti-rabbit secondary antibody (EMD Millipore) diluted 1:1,000 in blocking buffer for 1 h at room temperature. Vectastain Elite ABC and diaminobenzidine substrate kits were used for immunoperoxidase staining according to the manufacturer’s instructions (Vector Laboratories).

### Migration and invasion assays

Cells, grown to 90% confluence, were pretreated with 25 μg/ml mitomycin C (Sigma-Aldrich) for 1 h. A wound was created using a 2-mm pipette tip. Cells were rinsed briefly in PBS, complete medium was added, and migration into the wound was monitored at the indicated time points by imaging at × 40 magnification. Cell invasion was assessed using the Cell Invasion Assay Kit (Chemicon). Briefly, polycarbonate filters were coated with ECMatrix and placed in a transwell chamber. Medium, with or without 10% FBS, was added to the lower compartment of the chamber, then 3 × 10^5^ cells, suspended in DMEM without FBS, were added to the upper compartment. After 24 h incubation at 37 °C, the filters were fixed with methanol and the number of cells that had invaded through the basement membrane was counted.

### RNA sequencing analysis (RNA-seq.) and quantitative real-time PCR

For RNA seq. analysis, RNA libraries were prepared from 100 ng of total RNA from each sample according to the manufacturer’s instruction (www.lascience.co.kr), and sequenced on an Illumina HiSeq 2000 using TruSeq SBS sequencing software (version 3). Genes presenting at least twofold difference between groups with a *p* value <0.05 were considered differentially expressed.

Quantitative real-time PCR was performed using total cellular RNAs purified from cultured cells using Trizol reagent (Invitrogen) according to the manufacturer’s protocol. The extracted RNA samples were subsequently treated with MMLV reverse transcriptase (Promega). PCR products were analyzed on 1% or 1.2% agarose gels (Invitrogen) and analyzed using SsoFast EvaGreen Supermix (Bio-Rad). Quantification of gene expression was performed only in the linear range for each primer pair. The delta-delta cycle threshold (DDCT) method [31] was used to quantify changes in the expression of each specific gene normalized to the expression of the housekeeping gene *gapdh*. The sequences of primers used for amplification are provided in Additional file 2: Table S2.

### Western blot analysis

To prepare whole-cell extracts, cells were scraped from the dishes and suspended in protein extraction buffer (100 mM Tris-Cl (Sigma-Aldrich) pH 7.8, 10 mM NaCl (Sigma-Aldrich), 10% glycerol (Sigma-Aldrich), 1 mM sodium orthovanadate (Sigma-Aldrich), 50 mM sodium fluoride (Sigma-Aldrich), and 1 mM phenymethylsulfonyl fluoride (Sigma-Aldrich). Equal amounts of protein were separated by electrophoresis on 10% SDS-polyacrylamide gels, followed by electrophoretic transfer to nitrocellulose membranes (EMD Millipore). Membranes were blocked in 5% nonfat dry milk (Amresco LLC) and 0.1% Tween-20 (Amresco LLC) in Tris-buffered saline and probed with the following primary antibodies; the anti-ERK, anti-JNK, anti−p38, anti-phospho-JNK, anti-MEK, anti-FLAG, anti-p53, anti-p21, anti-GSK3β, and anti-ACTIN (Santa Cruz Biotechnology), anti-phospho-ERK, anti-phospho-p38, anti-phospho-GSK3β (Ser9), and anti-phospho-MEK (Cell Signaling). Antibody-antigen complexes were incubated with anti-rabbit or anti-mouse-IgG-peroxidase conjugates (Santa Cruz Biotechnology), followed by detection using an enhanced chemiluminescence (ECL) kit (GE Healthcare Life Sciences).

### Measurement of intracellular reactive oxygen species (ROS) and nitric oxide (NO)

For analysis of intracellular ROS levels, cells treated with 1 mM N-acetylcysteine (NAC; Sigma-Aldrich) for 1 h, and incubated with 10 μM 2′-7′-dichlorofluorescin diacetate (H_2_-DCFH-DA) (Invitrogen) for 30 min at 37 °C in the dark. Then, the cells were washed twice with PBS, analyzed by FACScan flow cytometer and Cell Quest 3.2 software (Becton, Dickinson and Company).

Intracellular NO was detected using an OxiSelect™ Intracellular Oxide Assay kit (Cell Biolabs Inc) according to the manufacturer’s instruction. In brief, 2 × 10^5^ cells were seeded in 6-well culture plates and grown for 24 h. Prior to the experiment, the cells were incubated with 1 mM L-NAME (NOS inhibitor, N(G)-nitro-l-arginine-methyl ester, Sigma-Aldrich) for 1 h. The cells were rinsed twice with PBS and then incubated for 2 h at 37 °C under 5% CO_2_ in the presence of the NO probe. After 2 h, the cells were lysed using lysis buffer and then the NO fluorescence was quantified using a SpectraFluor Plus device (Tecan Group Ltd.) set to excite at 485 nm and emit at 530 nm.

### Plasmid transfection

The pcDNA3-FLAG-tagged vectors containing either WT MEK or active MEK were introduced separately into CT + SS using Lipofectamine (Invitrogen). Stably expressing clones were selected using 1 μg/ml G418 (Calbiochem) for 20 days. The selected cells were assessed for Flag expression by western blot analysis.

### Statistical analysis

Data are shown as the mean ± standard error (SEM). Statistical analyses were performed using the two-tailed Student *t* test for two groups, in Excel (Microsoft, Redmond, WA, USA) or InStat 3 (GraphPad software, La Jolla, CA, USA). For the multiple comparison test, analysis of variance (ANOVA) was performed with Tukey-Kramer adjustment. A *p* value <0.05 was considered statistically significant.

## Results

### Hydrodynamic shear stress experienced during systemic circulation of tumor cells leads to acquisition of stemness and EMT potential

**Fig. 1:**
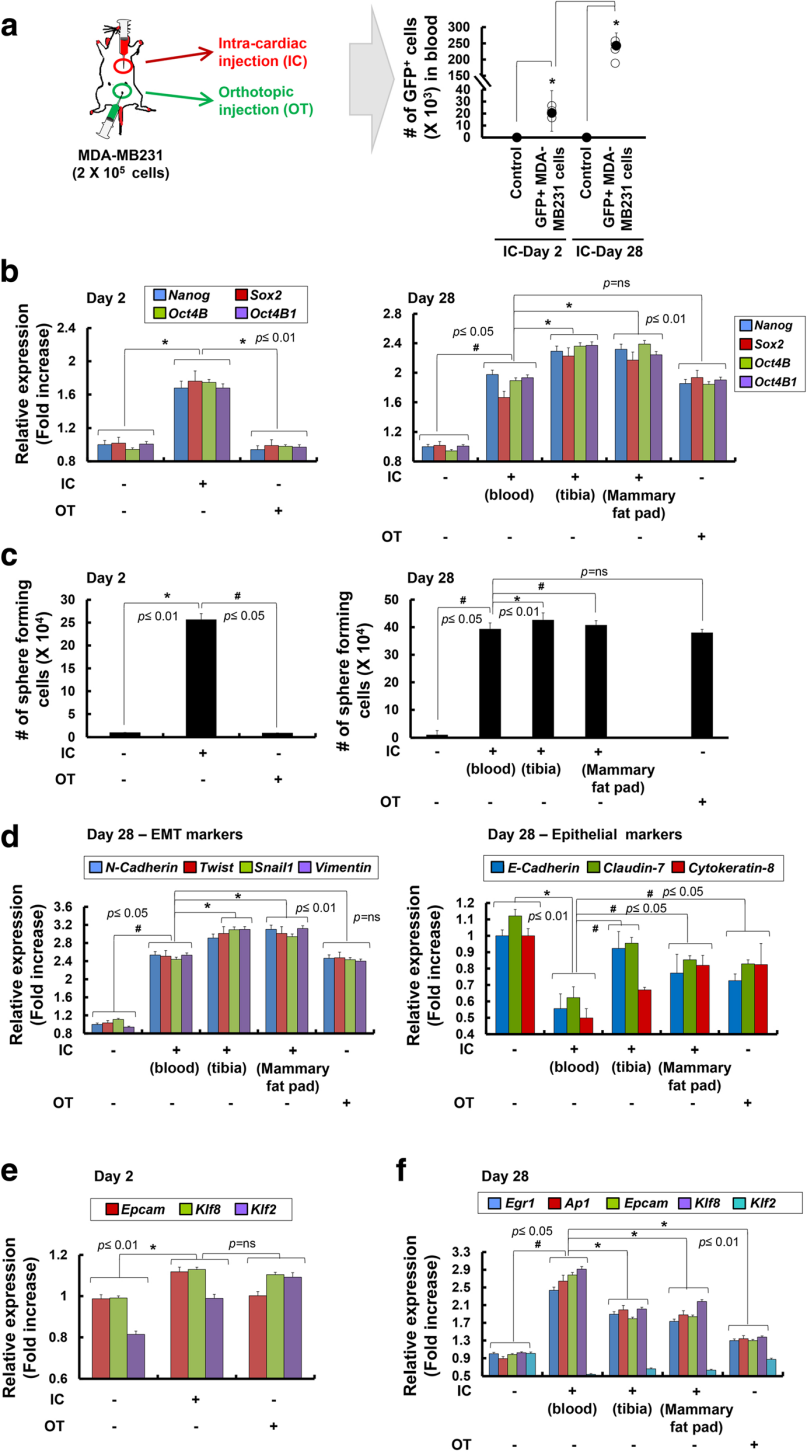
Analysis of tumor formation, transcriptional changes, and sphere-forming ability of MDA-MB231 cells harvested from the blood after intra-cardiac injection or from mammary fat pads after orthotopic injection. **a** Green fluorescent protein (GFP)^+^ MDA-MB231 cells (density, 2 × 10^5^ cells) were injected into the left ventricle of the heart or mammary fat pads of mice (*n* = 5). Right panel, the total bio-fluorescent GFP^+^ cells in the whole blood from the intra-cardiac (IC)-injected mice or PBS-injected control mice were isolated by fluorescence-activated cell sorting (FACS) and the number of GFP^+^ cells is presented. An average is shown as black circles; *p < 0.05. **b** Expression of stemness marker genes (*Nanog*, *Sox2*, *Oct4B*, and *Oct4B1*) was analyzed by quantitative real-time RT-PCR at 2 or 28 days following IC or orthotopic (OT) administration of MDA-MB231 cells (*n* = 5). In mice where MDA-MB231 cells were injected systemically (IC), secondary tumors formed in the tibia and mammary fat pads, and their expression of stemness marker genes was significantly increased (Tibia, Mammary fat pads). **c** Sphere-forming capacity of GFP^+^ MDA-MB231 cells harvested at 2 or 28 days from mice directly injected into left ventricle of the heart or orthotopically implanted into mammary fat pads (*n* = 5). **d** Expression of the epithelial-mesenchymal transition (EMT) marker (*N-Cadherin, Twist, Snail1,* and *Vimentin*; left panel) and epithelial marker (*E-Cadherin*, *Claudin-7*, and *Cytokeratin-8*) genes on GFP^+^ MDA-MB231 cells harvested at 28 days from blood, tibia, and mammary fat pads analyzed by quantitative real-time RT-PCR (*n* = 5). Expression of the shear stress (SS)-induced genes (*Egr1, Ap1, Epcam, Klf8,* and *Klf2*) on GFP^+^ MDA-MB231 cells harvested at 2 days (**e**) and 28 days (**f**) from blood, tibia, and mammary fat pads analyzed by quantitative real-time RT-PCR analysis (*n* = 5). Expression of each gene in quantitative real-time RT-PCR analysis was normalized to *Gapdh*. The data presented here are presented as mean ± SEM and are representative of three independent experiments. Statistically significant differences are tested at *p* < 0.05 significance

To initiate the metastatic spread of cancer, tumor cells are exposed to mechanical forces exerted by fluid SS, hydrostatic pressure, and tension [13, 16]. We hypothesized that SS applied to tumor cells during systemic blood circulation may trigger the transition of epithelial tumor cells into TICs, similar to that observed in hematopoietic stem cells (HSCs). To test this hypothesis, we injected GFP^+^ MDA-MB231 breast tumor cells directly into the left ventricles of the mice (Fig. 1a). Markedly elevated GFP signals were observed on day 28 after the injection, suggesting that CTCs remaining in blood circulation had undergone proliferation. The average number of bio-fluorescent GFP^+^ cells harvested from ~ 1 ml blood was 2.3 × 10^4^ cells on day 2 after the injection, which was approximately 12% of the total number of tumor cells (Fig. 1a). The number of GFP^+^ tumor cells in the blood increased to ~ 2.6 × 10^5^ cells by day 28 after the intra-cardiac injection. Importantly, circulating GFP^+^ tumor cells had significantly enhanced expression of *Nanog*, *Sox2*, and *Oct4* (*Oct4B* and *Oct4B1*) compared with those directly injected into the mammary fat pads (Fig. 1b). On day 28, expression levels of *Nanog*, *Sox2*, and *Oct4* in circulating GFP^+^ cells and cells injected into mammary fat pads (orthotopical (OT) injection) were similar, suggesting that static tumor cells acquire stemness property in the tumor microenvironment. More importantly, CTCs metastasizing to the tibia and the mammary fat pads at day 28 following intra-cardiac injection demonstrated even higher levels of all three stemness factors than those in circulation. These data suggest that CTCs had undergone epithelial-mesenchymal-like transition during circulation and that further stemness properties were acquired at the tumor site where the MET process culminated. Consistently, results of sphere formation assay showed that circulating GFP^+^ tumor cells formed more spheres than static GFP^+^ tumor cells harvested from the mammary fat pads (Fig. 1c, left panel). Moreover, GFP^+^ tumor cells harvested from the metastasized tibias and mammary fat pads of mice on day 28 had significantly greater sphere formation ability (Fig. 1c, right panel) and expression of EMT genes, including *N-cadherin*, *Twist*, *Snail1*, and *Vimentin*, than those in circulation or injected into the mammary fat pads (Fig. 1d left panel). Furthermore, expression of epithelial markers, such as *E-Cadherin*, *Claudin-7*, and *Cytokeratin-8*, were significantly decreased in circulation or injected into the mammary fat pads (Fig. 1d right panel). Expression of SS-induced genes, such as *Early growth response 1* (*Egr1*) [32], *Activator protein 1* (*Ap1*) [33], *Epithelial cell adhesion molecule (Epcam*) [34], and *Kruppel-like factor 8* (*Klf8*) [35], were the highest in the circulating GFP^+^ tumor cells (Fig. 1e and f). Although *Klf2* was reported to be one of the KLF family proteins the expression of which in vascular endothelium was induced by SS [36], its expression was not increased in circulating GFP^+^ tumor cells in the present study.

In contrast, marginal increase of SS-induced genes in static mammary tumors or those metastasized to the tibias and mammary fat pads were noted, indicating that CTCs are under the influence of severe SS. These data suggest that fluid SS experienced during systemic circulation of human breast tumor cells can lead to specific acquisition of mesenchymal stem cell (MSC)-like potential that promotes EMT, MET, and metastasis to distant organs.

### Shear stress induced by repeated shaking and suspension in vitro drives the conversion of MDA-MB231 tumor cells into high sphere-forming TICs

**Fig. 2:**
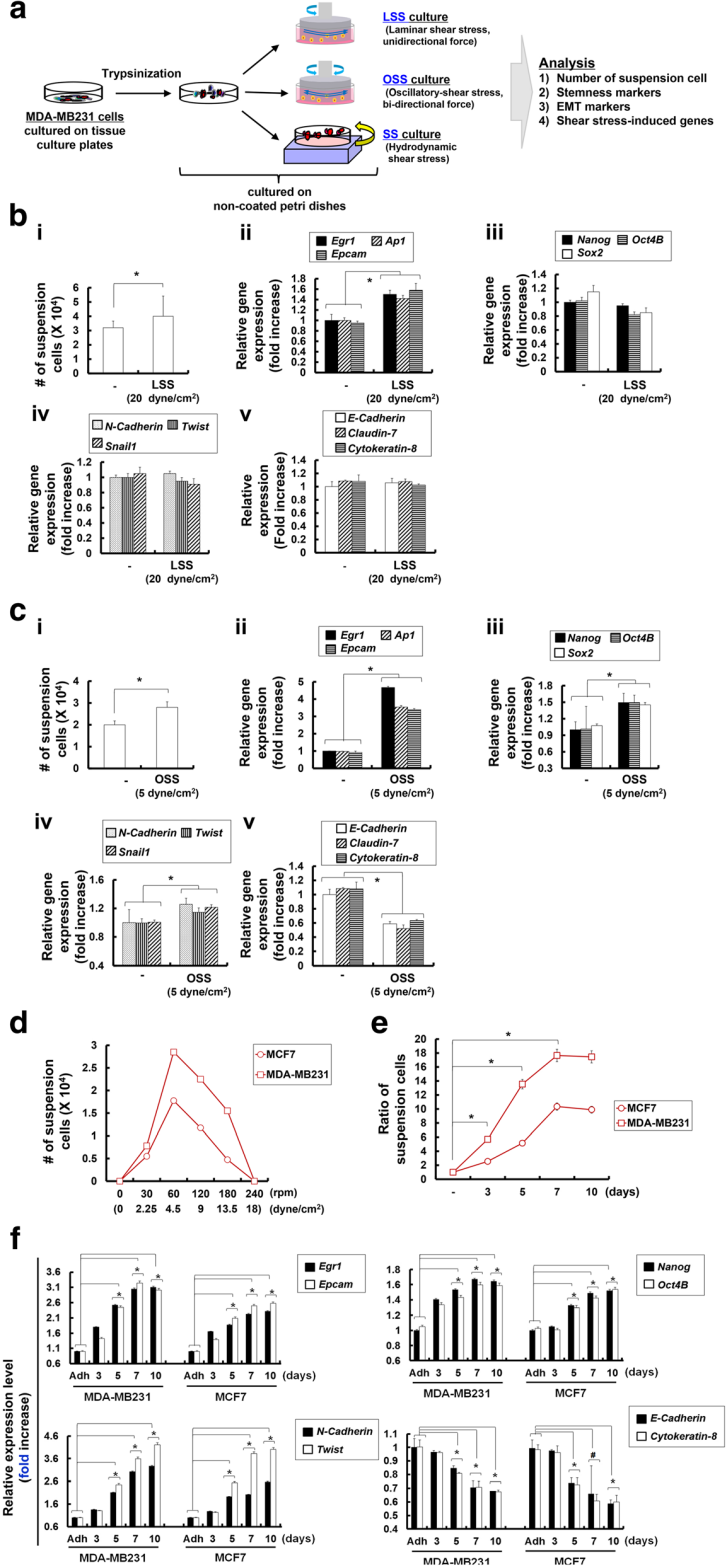
Hydrodynamic shear stress (SS) given as oscillatory shear stress (OSS) led to the acquirement of epithelial-mesenchymal transition (EMT) and stemness marker genes in MDA-MB231 cells. **a** Schematic illustration of in vitro fluid SS, including cone-and-plate viscometer-based laminar shear stress (LSS) or OSS and orbital shaker-based hydrodynamic SS. **b** (i) Number of suspension cells during LSS (270 rpm corresponds to 20 dyne/cm^2^) conditions of MDA-MB231 cells in non-coated Petri dishes after 24 h of culture. Cell viability was measured by trypan blue exclusion assay and error bars represent ± SD calculated from at least three independent experiments. Expression level of SS-induced (*Egr1*, *Ap1* and *Epcam* (ii)), stemness marker (*Nanog*. *Oct4B* and *Sox2*; (iii)), EMT marker (*N-Cadherin*, *Twist* and *Snail1*; (iv)), and epithelial marker (*E-Cadherin*, *Claudin-7*, and *Cytokeratin-8*; (v)) genes in cells in suspension during LSS conditions of MDA-MB231 cells in non-coated Petri dishes, analyzed by quantitative real-time RT-PCR. **c** (i) Number of suspension cells during OSS (67 rpm corresponds to 5 dyne/cm^2^) conditions in MDA-MB231 cells in non-coated Petri dishes after 24 h of culture. Expression level of SS-induced (*Egr1*, *Ap1* and *Epcam*; (ii)), stemness marker (*Nanog*. *Oct4B* and *Sox2*; (iii)), EMT marker (*N-Cadherin*, *Twist* and *Snail1*; (iv)), and epithelial marker (*E-Cadherin*, *Claudin-7*, and *Cytokeratin-8*; v) genes in suspension cells during OSS conditions of MDA-MB231 cells in non-coated Petri dishes, analyzed by quantitative real-time RT-PCR analysis. **d** Number of suspension cells during +SS (30–240 rpm corresponds to 2.25–18 dyne/cm^2^) conditions of breast cancer (MDA-MB231, MCF7) cells in non-coated Petri dishes after 24 h of culture. **e** Ratio of suspension cells in hydrodynamic SS (+SS) conditions (60 rpm corresponds to 4.5 dyne/cm^2^) of the indicated cells in non-coated Petri dishes were assessed on day 3, 5, 7, and 10. Cell viability was measured by trypan blue exclusion assay and error bars represent ± SD calculated from at least three independent experiments. **f** Expression of SS-induced (*Egr1* and *Epcam*), stemness marker (*Nanog* and *Oct4B*), EMT marker (*N-Cadherin* and *Twist*), and epithelial marker (*E-Cadherin*, *Claudin-7*, and *Cytokeratin-8*) genes expressed in the indicated cells over time on the indicated days, analyzed by quantitative real-time RT-PCR; ^**#**^*p* < 0.05, ^*^p < 0.01. Each gene expression in quantitative real-time RT-PCR analysis was normalized to *Gapdh*. The data presented here are presented as mean ± SEM and are representative of three independent experiments. Statistically significant differences were tested at *p* < 0.05 significance

We investigated whether SS given during blood circulation can be mimicked in vitro to induce the conversion of MDA-MB231 cells into TICs (Fig. 2a). Shear stress during blood circulation can be characterized as laminar, oscillatory, or hydrodynamic (Fig. 2a). At first, by using an in vitro fluid SS device, the cone-and-plate viscometer, we mimicked the SS exerted during a unidirectional steady flow (referred to as laminar shear stress (LSS)), which may be present in large straight segments of the arterial vasculature and is characterized by parallel layers of unidirectional flow with minimal disturbance between these layers [37]. Exposure of MDA-MB231 cells to high LSS (20 dyne/cm^2^) (Fig. 2b) led to a slight increase in the number of suspension cells (Fig. 2b-i) and expression of SS-induced genes (*Egr1*, *Ap1* and *Epcam*, Fig. 2b-ii). However, stemness factors (*Nanog*, *Oct4B*, and *Sox2*, Fig. 2b-iii), EMT markers (*N-Cadherin*, *Twist*, and *Snail1*, Fig. 2b-iv), and epithelial marker, *E-Cadherin*, *Claudin-7*, and *Cytokeratin-8*, Fig. 2b-v) showed no significant changes. Moreover, because bi-directional disturbed flow may correspond to blood flow in regions where arterial bifurcations and branch points are formed, which induce oscillating shear stress (OSS) [38], we also applied bi-directional disturbed flow to investigate the effect of OSS on CTCs. MDA-MB231 cells cultured under 5 dyne/cm^2^ OSS for 24 h showed a significant increase in the number of suspension cells (Fig. 2c-i) and expression of SS-induced (Fig. 2c-ii), stemness factor (Fig. 2c-iii), and EMT marker (Fig. 2c-iv) genes, compared with control cells not cultured under LSS. Moreover, expression of epithelial markers was significantly decreased upon OSS, compared with that of control cells (Fig. 2c-v).

In addition, as the SS given on the cells during blood circulation is reported to be within 2–14 dyne/cm^2^, which corresponds to 30–210 rpm of orbital shaking in vitro [39, 40], hydrodynamic shear stress (+SS) exerted during blood circulation was mimicked using an orbital shaker. Several breast cancer MCF7 and MDA-MB231 cells, were cultured in non-coated Petri dishes under orbital shaking at 30–240 rpm, which corresponds to 2.25–18 dyne/cm^2^. Among various SS tested, MDA-MB231 and MCF7 cells cultured at 60 rpm, corresponding to SS of 4.5 dyne/cm^2^, showed a significant increase in the number of suspension cells (Fig. 2d), reaching a plateau by 7 days following orbital shaking in culture (Fig. 2e). Furthermore, we observed that several different cancer cells, including breast cancer MCF7 and MDA-MB231 cells, hepatic cancer SNU447 and HepG2 cells, colon cancer HCT116 and HT29 cells, and pancreatic cancer Panc2 and Capan1 cells, showed an apparent increase in the number of suspension cells and, in particular, breast or hepatic cancer cells cultured at 60 rpm, corresponding to SS of 4.5 dyne/cm^2^, showed a significant increase in the number of suspension cells (Additional file 3: Figure S1). MDA-MB231 and MCF7 cells displayed a significant increase in the expression of marker genes for SS (*Epcam* and *Egr1*), stemness (*Nanog* and *Oct4B*), and EMT (*N-Cadherin* and *Twist*) (Fig. 2f). Moreover, these cells had decreased expression of epithelial marker (*E-Cadherin* and *Cytokeratin-8*) genes (Fig. 2f).

### +SS drives the conversion of primary breast cancer cells into CD24^low^/CD44^low^/CD133^high^TICs with elevated EMT marker expression

**Fig. 3:**
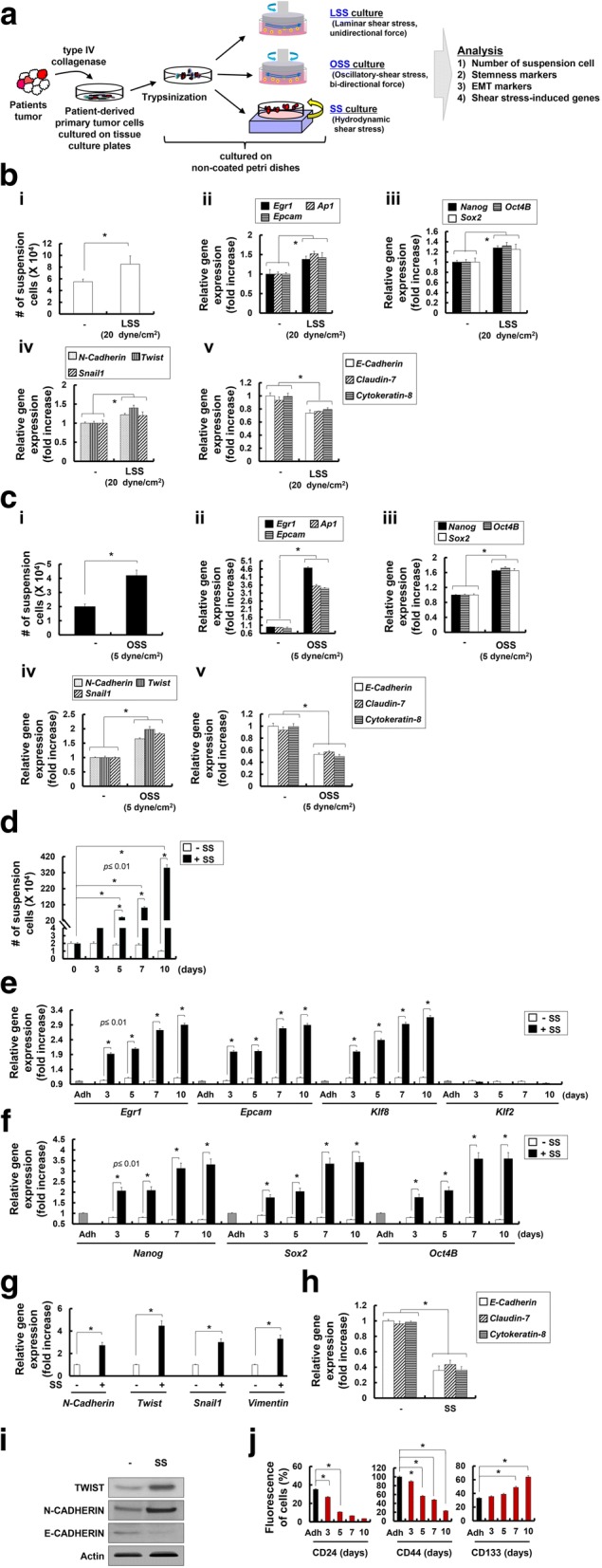
Hydrodynamic shear stress (+SS) given as orbital shaking led to the acquisition of epithelial to mesenchymal transition (EMT) and stemness-associated genes in primary epithelial tumor cells isolated from patients with breast cancer. **a** Schematic illustration of the in vitro fluid SS (laminar SS (LSS), oscillatory SS (OSS), or SS) that was exposed to breast cancer cells derived from chemotherapy-treated patients (CT-PCs). CT-PCs (density, 2 × 10^5^ cells) were isolated as described in “Methods” and subjected to cone-and-plate viscometer-based LSS or OSS and orbital shaker-based +SS in non-coated Petri dishes. **b** (i) Number of suspension cells during LSS (270 rpm corresponds to 20 dyne/cm^2^) conditions of CT-PCs at 24 h was measured by trypan blue exclusion assay and error bars represent ± SD calculated from at least three independent experiments. Expression level of SS-induced (*Egr1*, *Ap1* and *Epcam*; (ii)), stemness marker (*Nanog*. *Oct4B* and *Sox2*; (iii)), and EMT marker (*N-Cadherin*, *Twist* and *Snail1*; (iv)), and epithelial marker (*E-Cadherin*, *Claudin-7*, and *Cytokeratin-8*; **v**) genes in suspension cells during LSS conditions of CT-PCs in non-coated Petri dishes, analyzed by quantitative real-time RT-PCR. **c** (i) Number of suspension cells during OSS (67 rpm corresponds to 5 dyne/cm^2^) conditions of CT-PCs in non-coated Petri dishes after 24 h of culture. Expression level of SS-induced (*Egr1*, *Ap1* and *Epcam*; (ii)), stemness marker (*Nanog*. *Oct4B* and *Sox2*; (iii)), and EMT marker (*N-Cadherin*, *Twist* and *Snail1*; (iv)), and epithelial marker (*E-Cadherin*, *Claudin-7*, and *Cytokeratin-8*; (v)) genes in suspension cells during OSS conditions of CT-PCs in non-coated Petri dishes, analyzed by quantitative real-time RT-PCR analysis. **d** Number of cells in suspension without SS culture (-SS) or following SS culture (+SS) counted at 3, 5, 7, and 10 days using trypan blue exclusion assay. **e**-**h** Quantitative real-time RT-PCR analysis was performed on CT-PCs harvested at 3, 5, 7, and 10 days after culture to measure the expression of SS-induced (*Egr1*, *Epcam*, *Klf8* and *Klf2*) (**e**), stemness marker (*Nanog*, *Sox2*, and *Oct4B*) (**f**), EMT marker (*N-Cadherin*, *Twist, Snail1*, and *Vimentin*) (**g**) and epithelial marker (*E-Cadherin*, *Claudin-7*, and *Cytokeratin-8* (**h**) genes. **i** Western blot analysis was performed on CT-PCs (−) and CT-PCs harvested on 10 days after SS culture (SS) to detect the expression of EMT marker (TWIST and N-CADHERIN) and epithelial marker (E-CADHERIN) proteins. **j** Surface marker expression of CD24, CD44, and CD133 were analyzed using flow cytometry and presented as percentage (fluorescent^+^ cells/all cells × 100%). Expression of each gene in quantitative real-time RT-PCR analysis was normalized to *Gapdh*. The data are presented as mean ± SEM and are representative of three independent experiments. Statistically significant differences were tested at *p* < 0.05

To further demonstrate whether +SS simulated by repeated shaking and suspension promotes the acquisition of EMT potential among primary breast cancer cells, we harvested tumor cells (CT-PC) from primary sites in patients with advanced breast cancer who underwent neoadjuvant chemotherapy, and subjected cultures to LSS, OSS, and + SS (Fig. 3a). CT-PCs exposed to LSS showed a slight increase in the amount of suspension cells (Fig. 3b-i) and expression of SS-induced (Fig. 3b-ii), stemness marker (Fig. 3b-iii), or EMT marker (Fig. 3b-iv) genes. Moreover, these cells showed slight decrease in expression of epithelial marker (*E-Cadherin* and *Cytokeratin-8*) genes (Fig. 3b-v). Importantly, CT-PCs exposed to OSS, showed a significant increase in the number of suspension cells (Fig. 3c-i) and expression of SS-induced (Fig. 3c-ii), stemness factor (Fig. 3c-iii), and EMT marker (Fig. 3c-iv) genes, compared to cells not exposed to OSS. Furthermore, these cells showed significant decrease in expression of epithelial marker (*E-Cadherin* and *Cytokeratin-8*) genes (Fig. 3c-v). More importantly, CT-PCs exposed to +SS demonstrated a very significant increase in the number of suspension cells during 10-day culture (Fig. 3d), accompanied by substantial upregulation of SS-induced genes (*Egr1*, *Epcam*, and *Klf8*; Fig. 3e), stemness markers (*Nanog*, *Sox2*, and *Oct4B*; Fig. 3f), and multiple EMT markers, *N-cadherin*, *Twist*, *Snail1*, and *Vimentin* (Fig. 3g), compared with those cultured without shaking (-SS) and MDA-MB231 or MCF7 cells cultured under shaking (+SS in Fig. 2f). Upregulation of these stemness and EMT marker genes was evident from day 3 and plateaued by day 10 following application of +SS. Moreover, CT-PCs exposed to +SS showed significant decrease in expression of epithelial marker (*E-Cadherin* and *Cytokeratin-8*) genes (Fig. 3h). Furthermore, using western blot analysis, we confirmed the increased expression of EMT markers, TWIST and N-CADHERIN, and decreased expression of an epithelial marker, E-CADHERIN, in CT-PCs exposed to +SS, compared with those cultured without shaking (-SS) (Fig. 3i). Additionally, CT-PCs exposed to +SS showed significant decrease in expression of *p53* and *p21* genes (Additional file 4: Figure S2). CT-PCs displayed a CD24^middle^/CD44^high^/CD133^middle^ phenotype before +SS. However, after 10 days of +SS culture, they exhibited a CD24^low^/CD44^low^/CD133^high^ phenotype (Fig. 3j).

### CT-PCs generated upon +SS (CT + SS) demonstrate significantly increased tumorigenicity in vivo

**Fig. 4:**
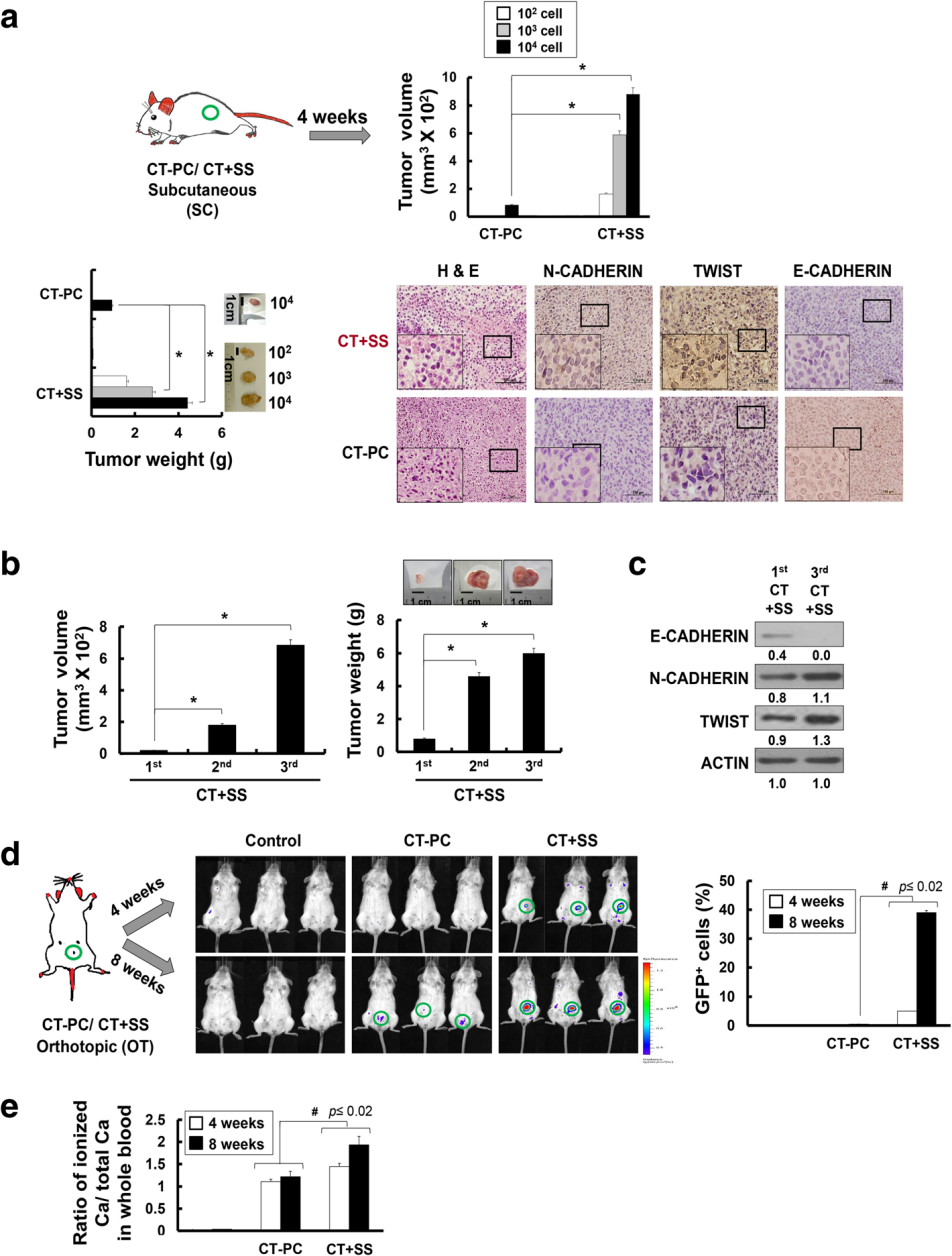
Patient-derived primary epithelial tumor cells cultured under hydrodynamic stress (SS) demonstrate significantly elevated in vivo tumorigenicity. **a** NOD/SCID mice were injected subcutaneously with increasing numbers of breast cancer cells derived from chemotherapy-treated patients CT-PCs or CT + SS, and tumor volumes (middle panel) and weights (right panel) were measured at 4 weeks after tumor cell inoculation. Representative images of tumors from the mice injected with the indicated number of cells (10^2^–10^4^ cells per site) are shown on the right (*n* = 3, *p < 0.05). Tumor tissues from the CT + SS-injected mice were analyzed by H&E staining or immunohistochemical analysis with the indicated antibody. Each indicated region (small squares) is magnified in the insets. **b** Tumor volumes and weights of successive passages of xenografts harboring CT + SS. The secondary xenograft was generated from injection of 50 cells isolated from the tumors formed from the preceding passage. Representative images of tumors harvested from mice are shown on the right (*n* = 4, *p < 0.05). **c** Protein expression of E-CADHERIN, N-CADHERIN, and TWIST was analyzed by western blot using the tumor tissues harvested from the 1^st^ and 3^rd^ passaged mice. Numbers refer to the densitometry analysis of each signal normalized against the corresponding anti-ACTIN values. **d** In vivo tumorigenicity of NOD/SCID mice injected orthotopically into the mammary fat pads with CT-PCs or CT + SS. Left panel, representative BFI images of mice at 4 and 8 weeks post-injection of the indicated cells (*n* = 3). Right panel, the percentage of green fluorescent protein (GFP)^+^ cells in the blood at 4 and 8 weeks post-injection was analyzed by flow cytometry. **e** The ratio of ionized calcium levels in whole blood from the CT-PCs- or CT + SS-injected mice were measured by the ABL800 analyzer. Error bars correspond to mean ± SD. Statistically significant differences were tested at *p* < 0.05

To investigate the tumorigenic potential of CT + SS, NOD/SCID mice were injected subcutaneously with increasing numbers of CT + SS or CT-PCs, and tumor sizes were measured at 4 weeks post implantation (Fig. 4a). Compared with parental CT-PCs, CT + SS implanted into the mice grew faster and had larger volume and weight, and the tumors developed even with as few as 100 implanted cells (Fig. 4a). Moreover, immuno-histological examination revealed strong N-CADHERIN and TWIST expression and low E-CADHERIN expression in mice bearing CT + SS cells (Fig. 4a). To further evaluate the serial tumorigenic potential of CT + SS in vivo [41, 42]*,* we subcutaneously injected 100 tumor cells derived from mice serially implanted with CT + SS into secondary and tertiary recipient NOD/SCID mice. CT + SS harvested from the secondary recipient mice formed tumors with as few as 50 implanted cells (data not shown). Moreover, cells harvested from the tertiary recipient mice formed approximately sixfold larger and heavier tumors (Fig. 4b) and had higher N-CADHERIN and TWIST expression and lower E-CADHERIN expression (Fig. 4c) than cells harvested from the primary recipient mice, confirming the in vivo regenerative capacity of CT + SS.

We further investigated whether similar stemness features could be preserved when tumor cells were orthotopically transplanted. GFP^+^ parental tumor (CT-PCs) or GFP^+^ CT + SS cells were injected into the mammary fat pads of NOD/SCID mice, and green fluorescence was monitored using bio-fluorescence imaging over time (Fig. 4d). After 4 weeks, tumor formation was evident in CT + SS-injected mice but not in CT-PC-injected mice. After 8 weeks, the number of GFP^+^ cells increased significantly in CT + SS-injected mice compared with that in CT-PC-injected mice (Fig. 4d). Moreover, ionized calcium levels in the whole blood [14, 43], a measure of metastasis, were significantly increased in CT + SS-injected mice (Fig. 4e). Collectively, these data strongly indicate that +SS is important for inducing the conversion of epithelial tumor cells into CSLCs/TICs with increased stemness and EMT marker expression, elevated sphere formation ability, and strong in vivo tumorigenicity.

### Increased ROS-responsive and NO-responsive gene transcription precedes the conversion of CT-PCs into CT + SS

**Fig. 5:**
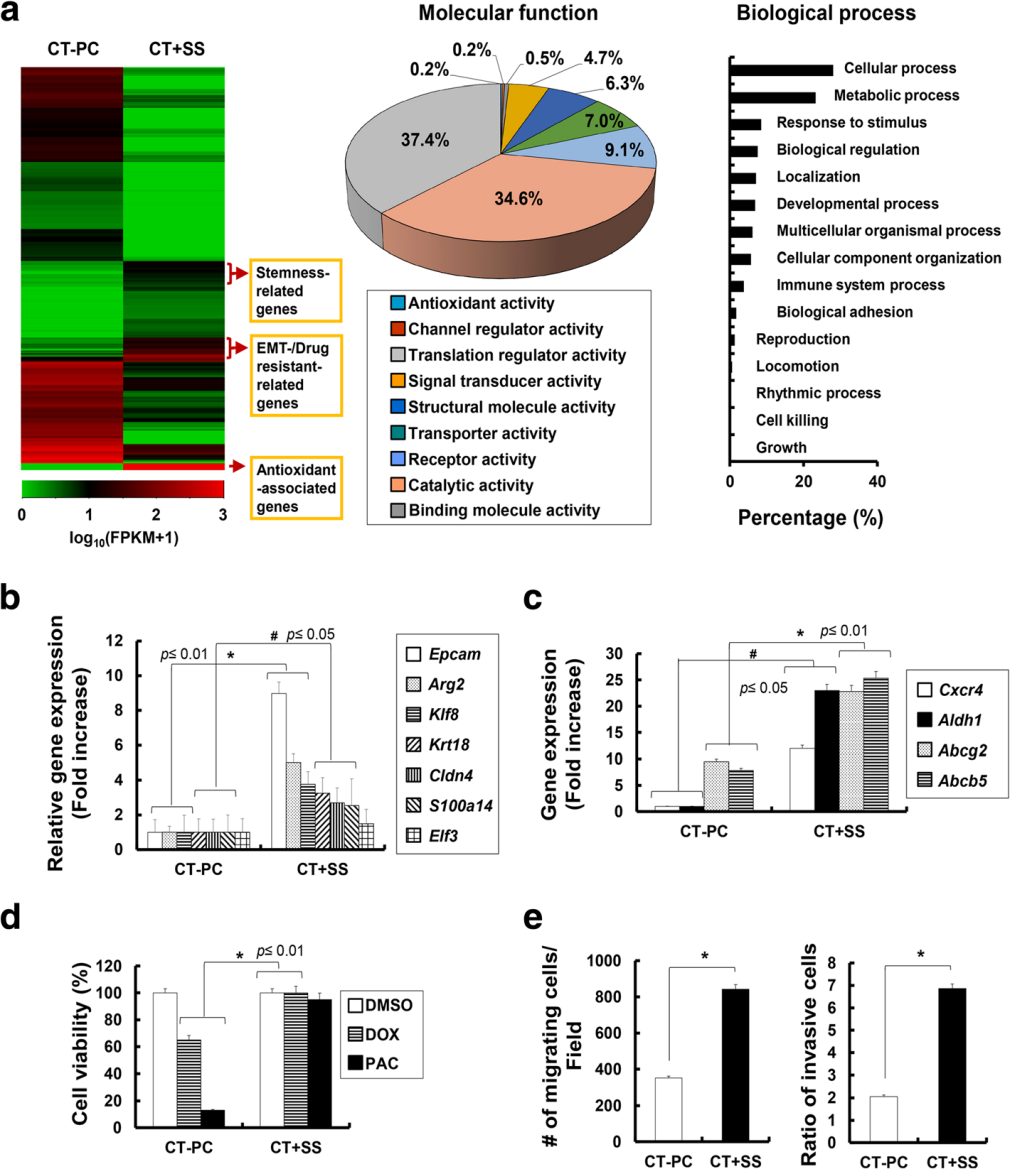
Patient-derived primary epithelial tumor cells acquire chemo-resistance with elevated migration and invasive potentials upon repeated orbital shaking. **a** Heatmap represents relative expression levels of genes showing least twofold difference between breast cancer cells derived from chemotherapy-treated patients (CT-PC) and CT + sheer stress (SS) with a *p* value < 0.05 is shown. Red and green represent the highest and lowest value of each gene analyzed, respectively. Gene Ontology (GO) analysis of RNA sequencing data revealed differential molecular function and biological processes on CT + SS compared with CT-PCs. GO categories for genes using the Network Ontology Analysis program (http://www.pantherdb.org/). RNA seq analysis of CT-PCs and CT-PCs under orbital shaking (CT + SS) for 10 days revealed upregulation of multiple SS-induced genes; *Epcam*, *Arg2*, *Cldn4*, *S100a14*, *Klf8*, and *Krt18* (**b**) and multi-drug resistance genes; *Cxcr4*, *Aldh1*, *Abcg2*, and *Abcb5* (**c**) upon +SS (*p* value compared with the non-shaking control group). **d** Cell viability of CT-PCs and CT + SS following treatment with doxorubicin or paclitaxel for 24 h (*p* value compared with dimethyl sulfoxide (DMSO)-treated cells). **e** Migration analysis was performed on CT-PCs and CT + SS by measuring the number of migrating cells per field of the wound made in the monolayer of cells. The migrating or invasive cells were measured in triplicate. Statistically significant differences were tested at *p* < 0.05

To understand molecular mechanism underlying the conversion of CT-PCs into CT + SS, we compared the global gene expression patterns in CT + SS with those in parental CT-PCs by performing RNA sequencing (RNA seq.). Heat map analysis and Gene Ontology (GO) data indicated that CT + SS showed altered expression of proteins involved in cellular/metabolic processes (biological process) and signal transduction (molecular function) and upregulated expression of stemness, EMT, drug-resistance, and antioxidant genes (Fig. 5 a). Moreover, CT + SS showed significantly increased expression of SS-induced genes, including *Epcam*, *Arg2*, *Cldn4*, *S100a14*, *Klf8*, and *Krt18* (Fig. 5b). Interestingly, these cells also showed upregulated expression of *Aldh1*, *Cxcr4* and multidrug resistance genes, including *Abcg2*, and *Abcb5* (Fig. 5c). Furthermore, CT + SS showed robust chemo-resistance to doxorubicin and paclitaxel (Fig. 5d) and elevated migration and invasion capacities (Fig. 5e). A similar gene expression pattern with elevated migration and chemo-resistance properties was observed in MDA-MB231 cells cultured under +SS (data not shown). These data suggest that +SS allows CT-PCs to acquire TIC characteristics, with a distinct CD133^high^ /CXCR4^high^/ALDH1^high^ phenotype.

**Fig. 6:**
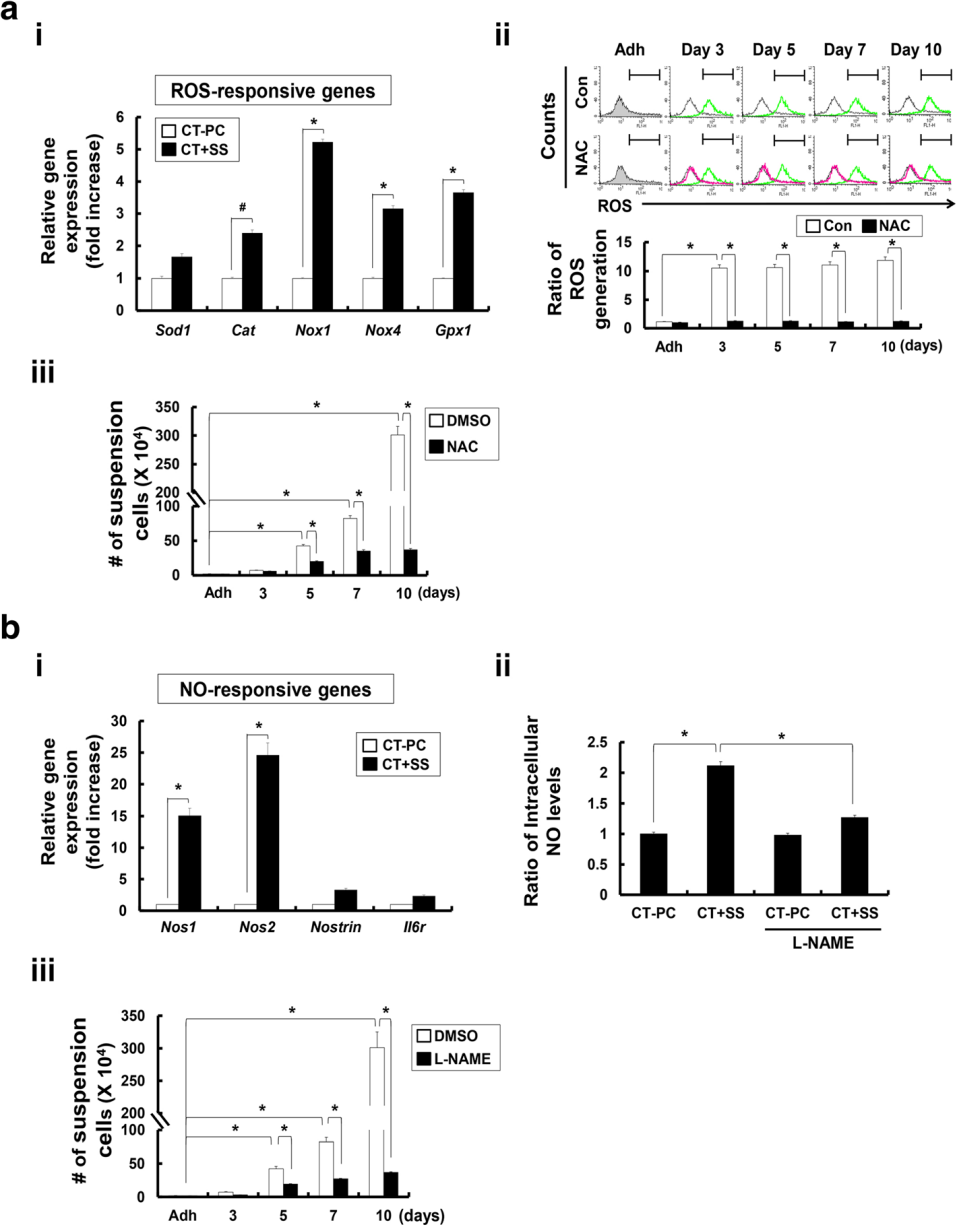
CT + shear stress (SS) demonstrate upregulation of reactive oxygen species (ROS)-responsive and nitric oxide (NO)-responsive genes. **a** Transcriptional profile of the selected ROS-responsive genes (*Sod1*, *Cat*, *Nox1*, *Nox4*, and *Gpx1*; i). (ii) Flow cytometry analysis of ROS generation of breast cancer cells derived from chemotherapy-treated patients (CT-PCs) treated with N-acetylcysteine (NAC) on day 3, 5, 7, and 10 during +SS (*p* value compared with adherent cells). iii, The number of suspended cells was counted on day 3, 5, 7, and 10 in the presence or absence of NAC treatment. **b** Transcriptional profile of the selected NO-responsive genes (*Nos1, Nos2*, and *Noxtrin*; (i)). Intracellular NO analysis was performed on CT-PC and CT + SS with 1 mM N(G)-nitro-l-arginine-methyl ester (L-NAME) by measuring the fluorescence NO probe (ii). The number of suspended cells was counted on day 3, 5, 7, and 10 in the presence or absence of L-NAME (iii). The data presented here are presented as mean ± SEM and are representative of three independent experiments. Statistically significant differences were tested at *p* < 0.05

Importantly, CT + SS showed increased transcription of ROS-responsive genes (*Sod1*, *Cat*, *Nox1*, *Nox4*, and *Gpx1*) and NO-responsive genes (*Nos1, Nos2*, and *Noxtrin*) (Fig. 6a-i and b-i). ROS functions in redox signaling and oxidative stress, which regulate diverse physiological parameters such as the growth factor stimulation and inflammatory response generation [44]. NO triggers a signaling pathway regulated by SS [45, 46] and hematopoiesis [47]. These results demonstrate that +SS can trigger generation of free radical species, such as ROS and reactive nitrogen species. To validate the results of RNA-seq., we measured intracellular ROS and NO levels in CT-PCs exposed to +SS. ROS level increased significantly in CT-PCs from day 3 of orbital shaking and remained elevated throughout the culture period (Fig. 6a-ii). This in turn activated ROS-responsive genes such as *Sod1*, *Cat*, *Nox1* and *Nox4* in CT-PCs from day 3 of repeated shaking (Fig. 6a-i). Treatment of CT + SS with the ROS scavenger N-acetylcysteine (NAC) suppressed ROS production (Fig. 6a-ii) and significantly reduced the number of proliferating CT-PCs (Fig. 6a-iii). NO content and NO-responsive gene (*Nos1* and *Nos2*) expression also increased in CT + SS (Fig. 6b-i). Importantly, treatment of CT + SS with a NOS inhibitor N(G)-nitro-l-arginine-methyl ester (L-NAME) decreased intracellular NO level (Fig. 6b-ii), thus decreasing their proliferation (Fig. 6b-iii). These data demonstrate that ROS-induced and NO-induced transcriptional changes under +SS are critical upstream events for the conversion of CT-PCs into highly proliferating CT + SS.

### Active suppression of ERK and GSK3β activities is a prerequisite for the generation of highly tumorigenic CT + SS

**Fig. 7:**
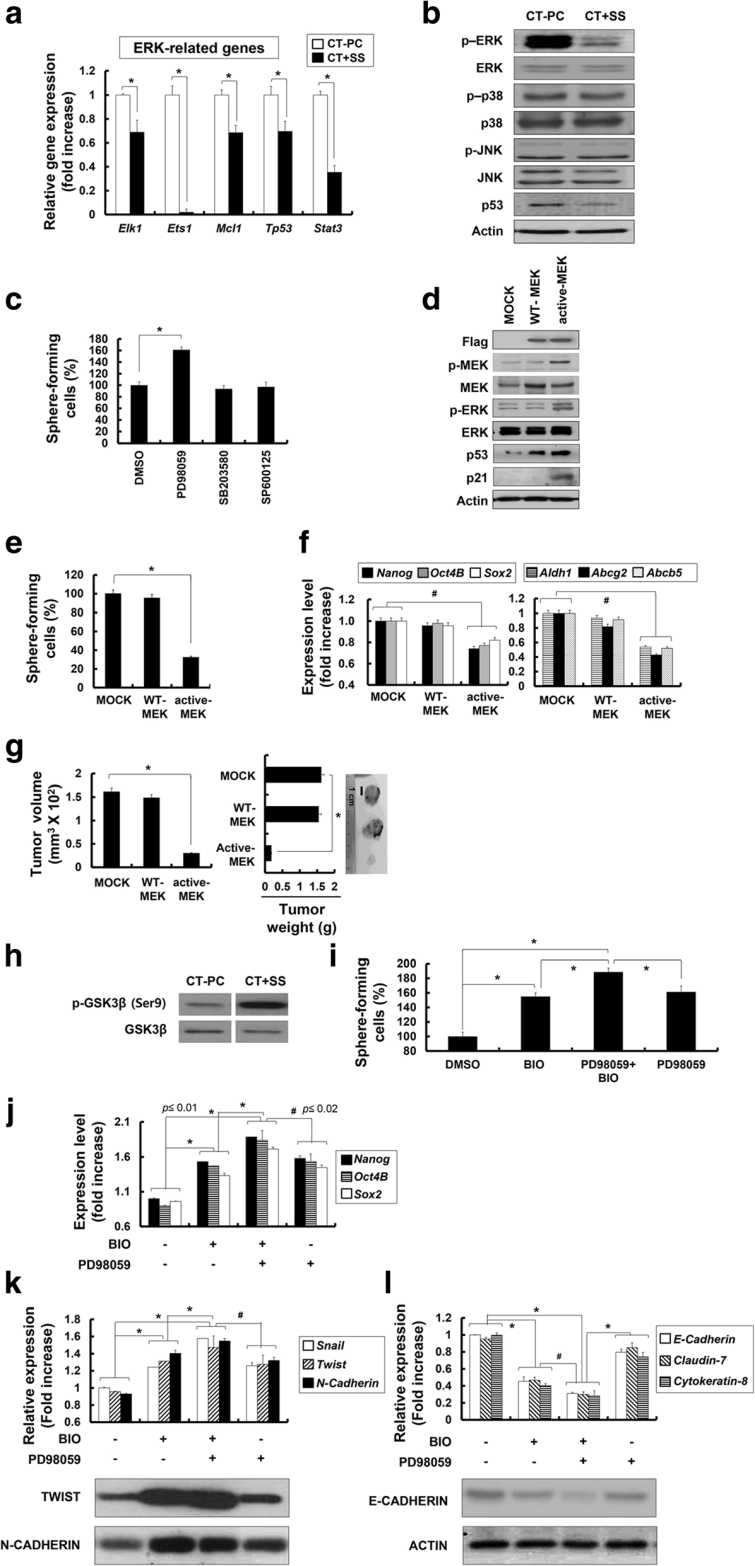
Downregulation of the extracellular signal-related protein kinase (ERK) pathway is critical for the conversion of breast cancer cells derived from chemotherapy-treated patients (CT-PCs) into CT + shear stress (SS) displaying cancer stem-like cell (CSLC)/tumor-initiating cell (TIC) properties. **a** Transcriptional profile of the selected ERK-related genes (*Elk1*, *Ets1*, *Mcl1*, *Tp53*, and *Stat3*). **b** Western blot analysis of p-ERK, ERK, p-p38, p38, p-JNK, JNK, and p53 from CT-PCs or CT + SS (10 days) n. **c** CT-PCs under +SS for 3 days were treated with PD98059 (mitogen-activated protein kinase (MEK) inhibitor), SB203580 (p38 MAPK inhibitor), and SP600125 (JNK inhibitor) for 24 h and subjected to sphere-formation assay (*p < 0.01 compared with dimethyl sulfoxide (DMSO)-treated cells). Error bars represent ± SD from the three independent experiments. **d** CT + SS obtained from 3-day culture under +SS were transfected with Flag-tagged wild-type MEK (WT-MEK) or active MEK (active-MEK), and the level of Flag, p-MEK, MEK, p-ERK, ERK, p53, and p21 were assessed by western blot. **e** Changes in the number of sphere-forming cells in WT-MEK or active-MEK-expressing CT-PCs are shown as percent changes (%) (*p < 0.01, compared to mock transfection). **f** Relative changes in the expression of self-renewal marker (*Nanog*, *Oct4B*, and *Sox2*) and multi-drug resistance (*Aldh1*, *Abcg2*, and *Abcb5*) genes are normalized to *Gapdh*. Data represent mean ± SD from three independent experiments (*p < 0.01). **g** In vivo tumorigenicity of mock, WT-MEK, and active-MEK carrying CT + SS implanted subcutaneously on NOD/SCID mice. Tumor volumes and weights were measured in NOD/SCID mice after inoculation with 2 × 10^5^ cells for 4 weeks. **h** Western blot analysis of p-GSK3β and GSK3β from CT-PCs or suspension CT + SS are shown. CT-PCs under +SS for 3 days were treated with PD98059 (MEK inhibitor) or/and BIO (GSK3β inhibitor) for 24 h and subjected to sphere-formation assay (**i**), quantitative real-time RT-PCR (**j** and upper panels of **k** and **l**), or western blot (lower panels of **k** and **l**) analyses of stemness (*Nanog*, *Oct4B*, and *Sox2*; **j**, EMT (*Snail*, *Twist*, and *N-Cadherin* (**k**)), and epithelial (*E-Cadherin*, *Claudin-7*, and *Cytokeratin-8*; **l**) marker genes. Relative changes in the expression of stemness and EMT marker genes are normalized to *Gapdh*; *p < 0.01, Error bars correspond to mean ± SD

Recent studies indicate that self-renewal of embryonic stem (ES) cells is induced by the downregulation of ERK-associated differentiation-inducing signaling pathways [48, 49]. Consistently, our RNA-seq. data showed downregulation of ERK-related genes (*Elk1*, *Ets1*, *Mcl1*, *Tp53*, and *Stat3*) in CT + SS (Fig. 7a). Moreover, CT + SS showed a significant reduction in ERK phosphorylation (Fig. 7b) but did not show any change in p38 MAPK and JNK phosphorylation levels. Moreover, the sphere-forming capacity of CT + SS under +SS culture increased after treatment with an MEK inhibitor PD98059 (Fig. 7c). Inhibition of p38 MAPK activity by SB203580 or JNK activity by SP600125 did not affect the sphere-forming capacity of CT + SS. To confirm the role of ERK in the conversion of CT-PCs into CT + SS, we introduced a FLAG-tagged WT MEK (WT-MEK) or constitutively active MEK (active-MEK) transgene into CT + SS (Fig. 7d). CT + SS overexpressing active-MEK showed a significant increase in MEK and ERK phosphorylation, which increased p53 and p21 expression (Fig. 7d). Furthermore, CT + SS overexpressing active-MEK showed dramatically reduced sphere formation capacity (Fig. 7e) and decreased stemness factors (*Nanog*, *Oct4B*, and *Sox2*) and drug resistance gene (*Aldh1*, *Abcg2*, and *Abcb5*) expression (Fig. 7f) compared with mock-transfected or WT-MEK-overexpressing cells. Importantly, CT + SS overexpressing active-MEK showed significantly decreased tumorigenicity in vivo (Fig. 7g) compared with mock-transfected or WT-MEK-overexpressing cells. These results confirm that suppression of the ERK pathway is critical for acquiring and maintaining CSLCs/TICs properties of CT + SS in vivo.

Recent studies demonstrate that downregulation of ERK activity is critical for the self-renewal of embryonic stem (ES) cells because ERK triggers the differentiation of these cells [48, 49]. Moreover, GSK3β inhibition consolidates biosynthetic capacity and suppresses residual differentiation [48]. Therefore, we examined whether CT + SS had a similar signaling cascade as that observed in ES cells by downregulating GSK3β. Indeed, we found that inhibitory phosphorylation at Ser9 position of GSK3β was increased in CT + SS (Fig. 7h), implying that GSK3β activity was suppressed in CT + SS upon +SS. Consistently, CT-PCs under +SS had increased sphere formation capacity after treatment with GSK3β inhibitor BIO (Fig. 7i). Furthermore, co-treatment of CT + SS with the MEK inhibitor, PD98059 and GSK3β inhibitor, BIO, synergistically increased their sphere formation capacity (Fig. 7i) and stemness factor (*Nanog*, *Oct4B*, and *Sox2*; Fig. 7j) and EMT marker (*Snail, Twist*, and *N-Cadherin*; Fig. 7k) expression. Consistently, we found that co-treatment of CT + SS with PD98059 and BIO synergistically increased both messenger RNA (mRNA) and protein expression of EMT marker (TWIST and N-CADHERIN) genes while decreasing that of epithelial markers (*E-Cadherin*, *Claudin-*7, and *Cytokeratin-8*) (Fig. 7l). Taken together, these data indicate that downregulation of ERK and GSK3β activities in CT + SS is critical for maintaining their EMT potential and tumorigenicity under +SS.

## Discussion

**Fig. 8:**
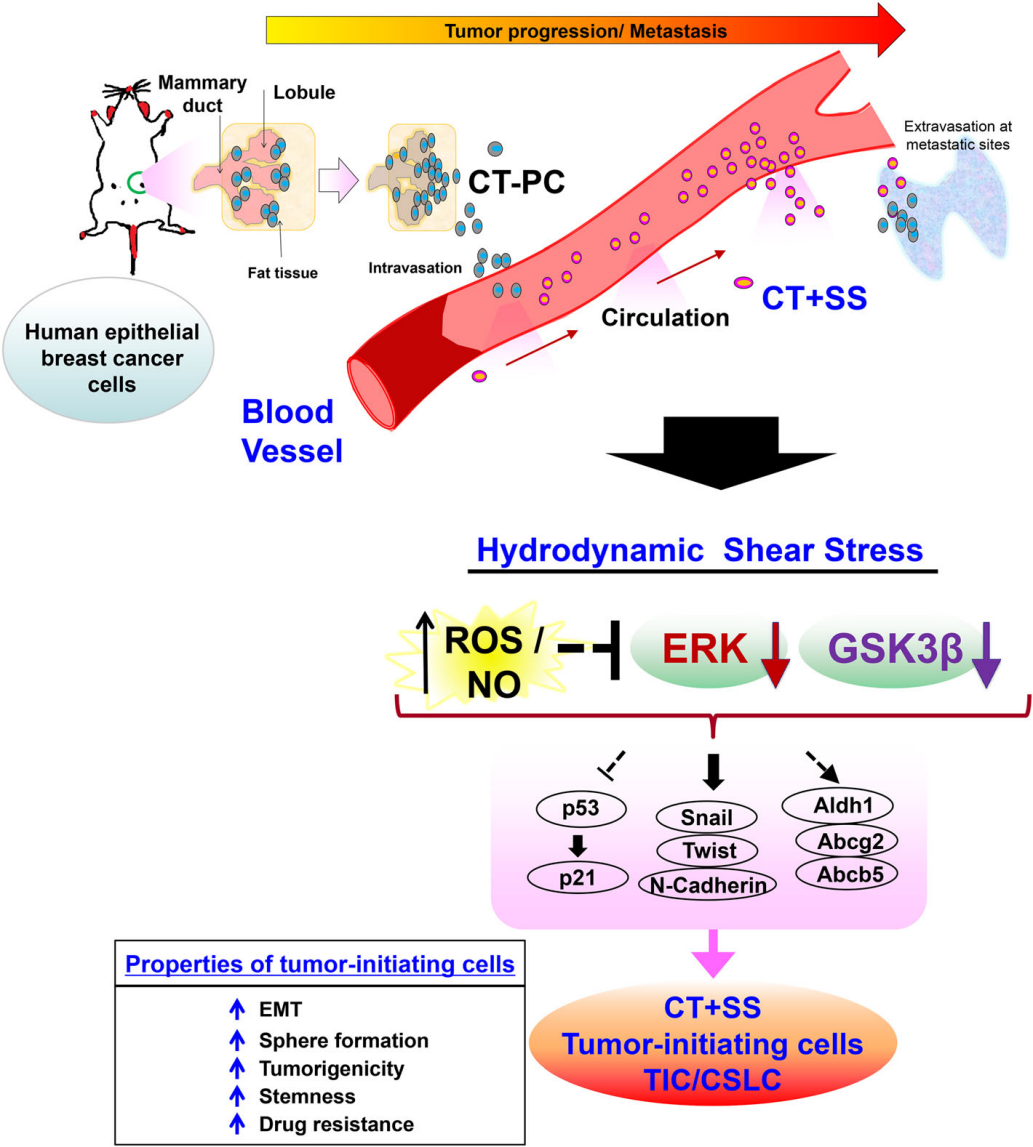
Role of the hydrodynamic shear stress (+SS) in the conversion of epithelial tumor cells into cancer stem-like cells (CSLCs)/tumor-initiating cells (TICs). Proliferating tumor cells near the periphery of solid tumor mass are translocated into nearby blood vessels due to the nature of loose mosaic vessels. Once translocated into blood vessels, epithelial tumor cells are constantly exposed to severe SS in the systemic circulation and prone to dying in the absence of a strong survival signal. +SS given to tumor cells generate reactive free radial species, reactive oxygen species (ROS) and nitric oxide (NO), and cause transcriptional changes in ROS-dependent and NO-dependent pathways along with multiple SS-associated gene pathways. These early signaling events lead to active suppression of extracellular signal-related protein kinase (ERK) and glycogen synthase kinase (GSK)3β pathways to confer plasticity on the epithelial tumor cells, and maintain undifferentiated mesenchymal stem cell properties. As a result, tumor cells acquire survival benefit within the blood circulation with elevated stemness, Epithelial to mesenchymal transition (EMT)/mesenchymal to epithelial transition (MET) properties, invasive properties, and multi-drug resistance phenotype. CT-PCs, breast cancer cells derived from chemotherapy-treated patients

EMT contributes to the early-stage dissemination of primary tumors and is a prerequisite for the invasion and metastasis of breast cancer cells. However, the importance of EMT in vivo is constantly under debate because metastatic lesions mostly exhibit epithelial phenotypes, presumably generated by MET, and that finding mesenchymal types of tumor cells from neighboring stromal cells has been difficult [50]. In fact, recent studies have challenged the current dogma by demonstrating that EMT may not be a prerequisite for metastasis [51, 52]. Cell lineage tracing studies indicate that metastatic tumor cells found in the lungs of MMTV-PyMT mice maintained their breast epithelial phenotype, suggesting that tumors of epithelial origin can enter nearby blood vessels without a pre-EMT requirement, migrate through the systemic circulation, and form secondary nodules in distant organs. The precise mechanism underlying these events is still not fully understood. However, our data provide evidence that +SS plays an important role in promoting the survival of circulating epithelial tumor cells by inducing lineage plasticity, which is characterized by the upregulated expression of stemness, EMT and epithelial markers (summarized in Fig. 8). Orbital shaking + SS or OSS, which may mimic arterial and venous circulation, sufficiently triggers this phenotypic transition in epithelial tumor cells. We demonstrate that +SS-exposed cells acquire stem-like properties and become CSLCs/TICs and that this +SS-induced TIC formation is dependent on the generation of ROS and NO, accompanied by downregulation of the ERK and GSK3β pathways.

During metastasis, tumor cells are exposed to mechanical forces induced by fluid SS, hydrostatic pressure, and tension. To enter the vascular microenvironment, cancer cells penetrate surrounding tissue and enter nearby blood and lymphatic vessels. Interstitial flow is the slow movement of fluid around the cells and through the pores of the ECM that comprise the interstitium. One of the main functions of interstitial flow is lymphatic drainage, which returns plasma from leaky capillaries back to the bloodstream. The velocities of interstitial flows are believed to range from 0.1 to 1.0 μm/sec in normal tissues. Hemodynamic shear forces in the bloodstream range from 0.5 to 4.0 dyne/cm^2^ in the venous circulation and 4.0 to 30.0 dyne/cm^2^ in arterial circulation. Cancer cells are primarily exposed to erythrocytes, leukocytes, and platelets upon entering the bloodstream, as studies have shown that in patients the concentration of cancer cells in the blood is on the order of one in a million leukocytes, or one in a billion blood cells. These hemodynamic shear stresses and velocities can affect the viability and metastatic potential of cancer cells. A recent study revealed that the fluid SS (0.05 dyne/cm^2^), which may correspond to the velocity of fluid flow in the interstitium, can promote cancer motility through modulating the Yes-associated protein (YAP1)-related ROCK-LIMK-cofilin signaling pathway [53]. Another recent study has demonstrated that circulation of hematopoietic stem cells can trigger the onset of hematopoiesis and embryogenesis [18]. The fluid SS (5 dyne/cm^2^) was shown to increase the expression of Runx1 in CD41^+^c-Kit^+^ hematopoietic progenitor cells with concomitant augmentation of their hematopoietic colony-forming potential. Our data showed that the +SS (4.5 dyne/cm^2^), which may mimic the hydrodynamic SS in the blood flow in the arterial vasculature, can trigger transition of epithelial breast tumor cells into stem-like CSLCs/TICs by upregulating stemness factor along with EMT factors primarily through modulation of ROS and NO generation, accompanied by downregulation of the ERK and GSK3β pathway. In our study, we could not detect significant increase in expression of YAP1 or Runx1 (data not shown). Although the detailed mechanism may be different, our data revealed that biomechanical forces appeared to be important micro-environmental factors that not only drive hematopoietic development but also lead to acquisition of CSLCs/TICs potential in cancer metastasis.

Interestingly, we found that the majority of CD24^middle^/CD44^high^/CD133^middle^/ CXCR4^low^/ALDH1^low^ primary parent tumor cells (CT-PCs) were converted to CD24^low^/CD44^low^/CD133^high^/CXCR4^high^/ALDH1^high^ TICs under +SS in vitro. Since expressions of CD133, CXCR4, and ALDH1 have been associated with multiple chemo-resistance [54, 55], acquisition of resistance to doxorubicin and paclitaxel in our CT + SS is due to upregulation of these genes upon +SS. Moreover, the fact that tumor cells introduced into the artery directly through intra-cardiac injection demonstrated higher stemness and EMT/MET potentials than those locally introduced into the mammary fat pads, further highlighting the importance of hydrodynamic force as a facilitator of CSLCs/TIC conversion. Upon serial passaging in vivo, CT + SS tumor cells obtained by +SS acquired cancer stem-like properties, showing tumor development with only 50 cells implanted.

CT-PCs converted to TICs could acquire SS-induced genes, such as *Egr1*, *Ap1*, *Epcam*, *Jun,* and several *Klf* family genes, upon +SS application, suggesting that stress-responsive signaling pathways are hypersensitive in CT + SS cells compared with their parental tumor cells. SS-inducible genes such as the *Egr1* drive expression of *Ap1*, a transcription factor composed of protein dimers of c-JUN and c-FOS, which contributes to EPCAM-dependent breast cancer invasion [56]. KLF8, which was also induced by SS, is expressed in several human cancers and is known to repress E-Cadherin transcription, thereby augmenting the motility and invasiveness of cancer cells [35]. Taken together, our findings suggest that upregulation of these stress-responsive genes may trigger the transition from primary parental tumor cells to more highly invasive tumor-initiating cells.

It is notable that the shear stress-dependent TIC formation of breast tumor cells depends on ROS/NO generation and downregulation of the ERK and GSK3β pathways. Activation of ERK/MAPK pathways is a key event in cell proliferation and tumor progression, that occurs downstream of pathways associated with several growth factors including EGF, PDGF, VEGF, FGF, and insulin, etc. [57–62]. Therefore, it was surprising to find that in this study TIC conversion required suppression of ERK activation. However, recent reports show that self-renewal of ES cells does not require growth factor signaling, but is enabled solely by downregulation of differentiation-inducing signaling pathways mediated by MAPK [48, 49]. Additionally, inhibition of GSK3β consolidates biosynthetic capacity and suppresses residual differentiation. ES cells cultured in defined medium with inhibitors of two kinases (MEK and GSK3β), a condition known as “2i”, are thought to represent a naive ground state of ES cells. Since, we also observed downregulation of GSK3β activities by increased p-GSK3β (Ser9) in CT + SS cells and upregulation of Wnt/β-catenin signal pathways, stemness of CT + SS cells appeared to be actively maintained by “2i” states. Therefore, the ERK inhibition seen in CT + SS cells cultured under orbital shaking may establish conditions required to maintain their stem-like ground states by active suppression of ERK, similar to that operating in ES cells. Furthermore, orbital shaking of primary tumor cells facilitated gradual loss of *p53*, one of the driver genes mutated in tumor progression [63]; accumulation of WT *p53* in the nucleus causes direct inhibition of *Nanog* expression in mouse ES cells [64] and suppression of *p53* expression leads to de-repression of *Nanog,* which is required for maintenance of mouse ES cell self-renewal. Although detailed mechanisms of epithelial-TIC conversion by hydrodynamic forces awaits more thorough biochemical investigation, *p53* loss in TICs may affect upregulation of *Nanog* and other self-renewal marker genes.

## Conclusions

We provide both in vivo and in vitro evidence that the conversion from epithelial tumor cells into specific high sphere-forming CD24^low^/CD44^low^/CD133^high^/CXCR4^high^/ALDH1^high^ CSLCs/TICs can occur upon hydrodynamic shear stress experienced during systemic circulation and the conversion is dependent on ROS/NO generation and suppression of ERK/GSK3β, a mechanism similar to that operating in embryonic stem cells to prevent their differentiation while promoting self-renewal. Our data highlight previously neglected “shear stress” forces as a critical factor in promoting the conversion of circulating tumor cells to CSLCs/TICs within the blood circulation and, in endowing the plasticity required for epithelial tumor cells to maintain self-renewal signaling pathways.

## Additional files

Additional file 1: **Table S1.** Clinical characteristics of the patients with breast cancer in this study. (PDF 39 kb)

Additional file 2: **Table S2.** Sequences of the primers used in this study. (PDF 20 kb)

Additional file 3: **Figure S1.** The number of suspension cells during +SS conditions of hepatic, colon, and pancreatic cancer cell lines were assessed. Left panel, number of suspension cells during +SS (30–240 rpm corresponds to 2.25–18 dyne/cm^2^) conditions of hepatic cancer (SNU447, HepG2), colon cancer (HCT116, HT29), and pancreatic cancer (Panc2, Capan1) cells in non-coated Petri dishes were shown after 24 h of culture. Right panel, ratio of suspension cells +SS conditions (60 rpm corresponds to 4.5 dyne/cm^2^) conditions of indicated cells in non-coated Petri dishes were assessed on day 3, 5, 7, and 10. (PDF 81 kb)

Additional file 4: Figure S2 Quantitative real-time RT-PCR analysis was performed on indicated cells, at days indicated, to measure the expression of *p53* and *p21* genes expressed in the indicated cells over time; ^*^p < 0.01. (PDF 36 kb)

## Abbreviations

AP1: Activator protein 1
CLSC: Cancer-like stem cell
CTC: Circulating tumor cell
CT-PCs: Breast cancer cells derived from chemotherapy-treated patients
DMEM: Dulbecco’s modified Eagle’s medium
DMSO: Dimethyl sulfoxide
ECM: Extracellular matrix
EGR1: Early growth response protein 1
EMT: Epithelial to mesenchymal transition
EPCAM: Epithelial cell adhesion molecule
ERK: Extracellular signal-related protein kinase
ES: Embryonic stem
FBS: Fetal bovine serum
GFP: Green fluorescent protein
GO: Gene Ontology
GSK: Glycogen synthase kinase
H&E: Hematoxylin and eosin
JNK: c-Jun N-terminal kinase
KLF8: Kruppel-like factor 8
LSS: Laminar shear stress
MAPK: Mitogen-activated protein kinase
MET: Mesenchymal to epithelial transition
MSC: Mesenchymal stem cell
NAC: N-acetylcysteine
NO: Nitric oxide
OSS: Oscillatory shear stress
OT: Orthotopical
PBS: Phosphate-buffered saline
RBC: Red blood cells
ROS: Reactive oxygen species
SS: Shear stress
TIC: Tumor-initiating cell
WT: Wild-type
