# Supplementary material for: Hydrodynamic shear stress promotes epithelial-mesenchymal transition by downregulating ERK and GSK3β activities
Source: Breast Cancer Res. 2019 Jan 16;21:6. doi: 10.1186/s13058-018-1071-2 (PMC6335853; doi:10.1186/s13058-018-1071-2)
Supplement: Supplementary file 1 — Table S1. Clinical characteristics of the patients with breast cancer in this study. (PDF 39 kb) [file 13058_2018_1071_MOESM1_ESM.pdf]

Additional file 1

Table S1 Clinical characteristics of the breast cancer patients in this study.

Patient number (IRB number)	Age	Histology Grade	Diagnosis	Tumor size	Chemo-therapy	HER2	KI-67 (%)	p53 (%)	ER (%)	PR (%)
1 (1)	45	3	IDC	3.5	X	NA	0	0	80	82
2 (2)	49	4	IDC	4.5	X	NA	1	0	95	80
3 (3)	54	1	IDC	1.3	X	NA	20	95	0	0
4 (4)	68	1	IDC	1.1	X	NA	10	80	95	5
5 (5)	41	2	IDC	2	X	NA	15	1	80	84
9 (6)	42	1	Fibroadenoma	0.7	X	NA	0	0	0	0
11 (7)	65	3	IDC	3.1	X	NA	5	10	10	10
12 (8)	74	1	IDC	1.5	X	NA	5	10	80	50
16 (9)	46	1	IDC	1.1	X	NA	20	50	50	0
18 (10)	70	3	IDC	3.3	O	NA	40	1	0	10
19 (11)	39	1	IDC	1.2	X	NA	10	0	90	90
21 (12)	43	2	IDC	2.6	X	NA	50	95	10	0
24 (13)	71	4	IDC	5.5	X	NA	10	1	90	10
25 (14)	64	1	IDC	0.8	X	NA	5	0	95	10
27 (15)	38	4	Ductal carcinoma	4.9	X	NA	100	0	0	90
28 (16)	53	3	IDC	4	O	NA	0	0	75	75
31 (17)	36	2	IDC	2	X	O	50	1	60	0
32 (18)	46	2	IDC	2.3	X	O	80	100	0	0
33 (19)	49	3	IDC	3.3	O	NA	30	10	90	1
34 (20)	47	4	Phyllodes	7	X	NA	0	0	0	0
35 (21)	31	4	Ductal carcinoma	5.5	X	O	80	100	0	0
44 (22)	40	1	Fibroadenoma	0.8	X	O	0	0	0	0
45 (23)	49	2	IDC	2.2	X	O	0	0	70	95
