# Supplementary material for: Hydrodynamic shear stress promotes epithelial-mesenchymal transition by downregulating ERK and GSK3β activities
Source: Breast Cancer Res. 2019 Jan 16;21:6. doi: 10.1186/s13058-018-1071-2 (PMC6335853; doi:10.1186/s13058-018-1071-2)
Supplement: Supplementary file 2 — Table S2. Sequences of the primers used in this study. (PDF 20 kb) [file 13058_2018_1071_MOESM2_ESM.pdf]

Additional file 2

Table S2 Sequences of the primers used in this study.

Marker	GENE	Forward sequence	Reverse sequence
Stemness	*Nanog*	ATACCTCAGCCTCCAGCAGA	GCAGGACTGCAGAGATTCCT
	Sox2	ACACCAATCCCATCCACACT	GCAAGAAGCCTCTCCTTGAA
	Oct4A	CAGTGCCCGAAACCCACAC	GGAGACCCAGCAGCCTCAAA
	Oct4B	AGAACCGAGTGAGAGGCAAC	TGAGAAAGGAGACCCAGCA
	Oct4B1	GCACTTCTACAGACTATTCCTTG	TGATCCTCTTCTGCTTCAGG
EMT	*N-Cadherin*	GACAATGCCCCTCAAGTGTT	CCATTAAGCCGAGTGATGGT
	Twist	GCAAGCGCGGCAAGAAGTCT	GGGGTGCAGCGGCGCGGTC
	Snail1	TTTACCTTCCAGCAGCCCTA	CCCACTGTCCTCATCTGACA
	Vimentin	GACCCTGCAGTCATTCAGACA	GATTCCACTTTCCGTTCAAGGT
Epithelial	*E-Cadherin*	CAGCACGTACACAGCCCTAA	ACCCACCTCTAAGGCCATCT
	Claudin-7	TGCAGGCCACTCGAGCCCTA	GGCCTTTGTCCGGCACCCTG
	Cytokeratin-8	CAACTACATGGTTTACATGTTC	GCCAGTGGACTCCACGAC
Shear stress	*EGR1*	CTTCAACCCTCAGGCGGACA	GGAAAAGCGGCCAGTATAGGT
	AP1	AGCCCAAACTAACCTCACG	TGCTCTGTTTCAGGATCTTGG
	EPCAM	GGGAAATAGCAAATGGACACA	CGATGGAGTCCAAGTTCTGG
	KLF8	CATGAGTTCTGGACACTTCAGG	AGTGACCTGCTTGAATACCTG
	KLF2	CCTACACCAAGAGTTCGCATC	TGTGCTTTCGGTAGTGGC
Drug resistance	*ALDH1*	TGTTAGCTGATGCCGACTTG	AATCTGAGGGCCTTGAGTGA
	ABCG2	TTATCCGTGGTGTGTCTGGAG	TCCTGCTTGGAAGGCTCTATG
	ABCB5	GAGAGACAGTCGCCTTGGTC	CCACGATTGTAGTCCGACCT
ROS	*SOD1*	AGCCCAAACTAACCTCACG	TGCTCTGTTTCAGGATCTTGG
	CAT	CAGATAGCCTTCGACCCAAG	GTAGGGACAGTTCACAGGTAT
	NOX1	CCTGAGTCTTGGAAGTGGATC	ACGCTTGTTCATCTGCAATTC
	NOX4	TCACAGAAGGTTCCAAGCAG	ACTGAGAAGTTGAGGGCATTC
NO	*NOS1*	TGTTGAATCGGACCTTGTAGC	TGTAGTTGAGGTCAATGAAGG
	NOS2	GTTTGACCAGAGGACCCAG	ATCTCCTTTGTTACCGCTTCC
House keeping	*GAPDH*	ACATCGCTCAGACACCATG	TGTAGTTGAGGTCAATGAAGG
