# Supplementary material for: Hydrodynamic shear stress promotes epithelial-mesenchymal transition by downregulating ERK and GSK3β activities
Source: Breast Cancer Res. 2019 Jan 16;21:6. doi: 10.1186/s13058-018-1071-2 (PMC6335853; doi:10.1186/s13058-018-1071-2)

Additional file 3

Figure S1

Left panel, The number of suspension cells during +SS (30 - 240 rpm corresponds to 2.25 - 18 dyne/cm^2) conditions of hepatic cancer (SNU447, HepG2), colon cancer (HCT116, HT29), and pancreatic cancer (Panc2, Capan1) cells in non-coated petri-dishes were shown after 24 h of culture. Right panel, The ratio of suspension cells +SS conditions (60 rpm corresponds to 4.5 dyne/cm^2) conditions of indicated cells in non-coated petri-dishes were assessed on day 3,5,7, and 10. Cell viability was measured by trypan blue exclusion assay and error bars represent ± SD calculated from at least three independent experiments.

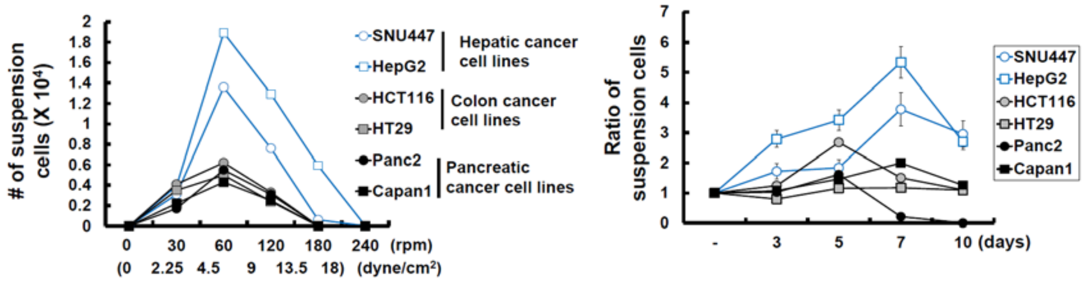

Supplement: Supplementary file 3 — Figure S1. The number of suspension cells during +SS conditions of hepatic, colon, and pancreatic cancer cell lines were assessed. Left panel, number of suspension cells during +SS (30–240 rpm corresponds to 2.25–18 dyne/cm2) conditions of hepatic cancer (SNU447, HepG2), colon cancer (HCT116, HT29), and pancreatic cancer (Panc2, Capan1) cells in non-coated Petri dishes were shown after 24 h of culture. Right panel, ratio of suspension cells +SS conditions (60 rpm corresponds to 4.5 dyne/cm2) conditions of indicated cells in non-coated Petri dishes were assessed on day 3, 5, 7, and 10. (PDF 81 kb) [file 13058_2018_1071_MOESM3_ESM.pdf]
