# Supplementary material for: Hydrodynamic shear stress promotes epithelial-mesenchymal transition by downregulating ERK and GSK3β activities
Source: Breast Cancer Res. 2019 Jan 16;21:6. doi: 10.1186/s13058-018-1071-2 (PMC6335853; doi:10.1186/s13058-018-1071-2)

Additional file 4

Figure S2

Quantitative real-time RT-PCR analysis was performed on indicated cells, at days indicated, to measure the expression of apoptosis marker (*p53* and *p21*) genes expressed in the indicated cells over time. *p<0.01.

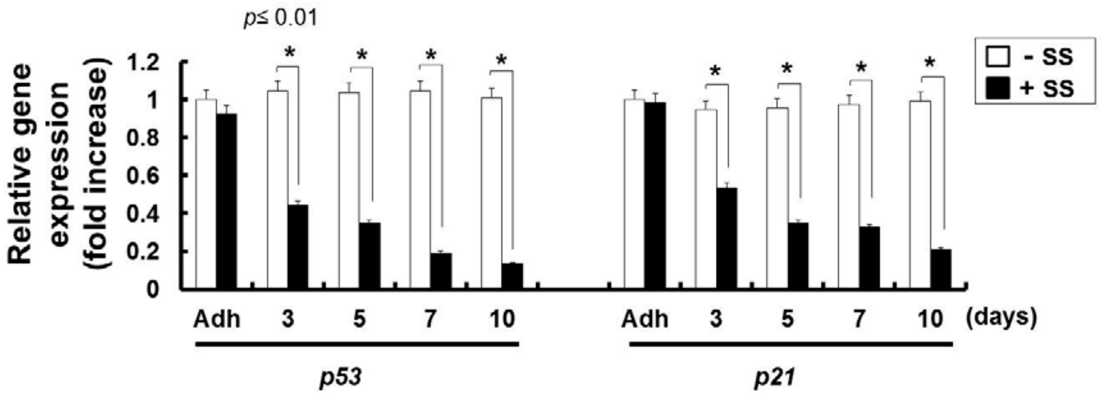

Supplement: Supplementary file 4 — Figure S2 Quantitative real-time RT-PCR analysis was performed on indicated cells, at days indicated, to measure the expression of p53 and p21 genes expressed in the indicated cells over time; *p < 0.01. (PDF 36 kb) [file 13058_2018_1071_MOESM4_ESM.pdf]
